# Accuracy and reliability of diffusion imaging models

**DOI:** 10.1016/j.neuroimage.2022.119138

**Published:** 2022-03-23

**Authors:** Nicole A. Seider, Babatunde Adeyemo, Ryland Miller, Dillan J. Newbold, Jacqueline M. Hampton, Kristen M. Scheidter, Jerrel Rutlin, Timothy O. Laumann, Jarod L. Roland, David F. Montez, Andrew N. Van, Annie Zheng, Scott Marek, Benjamin P. Kay, G. Larry Bretthorst, Bradley L. Schlaggar, Deanna J. Greene, Yong Wang, Steven E. Petersen, Deanna M. Barch, Evan M. Gordon, Abraham Z. Snyder, Joshua S. Shimony, Nico U.F. Dosenbach

**Affiliations:** a Department of Psychiatry, Washington University School of Medicine, St. Louis, MO 63110, United States of America; b Department of Neurology, Washington University School of Medicine, St. Louis, MO 63110, United States of America; c Mallinckrodt Institute of Radiology, Washington University School of Medicine, St. Louis, MO 63110, United States of America; d Department of Neurological Surgery, Washington University School of Medicine, St Louis, MO 63110 United States of America; e Department of Chemistry, Washington University in St Louis, St. Louis, MO 63110, United States of America; f Kennedy Krieger Institute, Baltimore, MD 21205, United States of America; g Department of Neurology, Johns Hopkins University School of Medicine, Baltimore, MD 21287, United States of America; h Department of Pediatrics, Johns Hopkins University School of Medicine, Baltimore, MD 21287, United States of America; i Department of Cognitive Science, University of California, San Diego, La Jolla, CA, United States of America; j Department of Obstetrics and Gynecology, Washington University School of Medicine, St. Louis, MO 63110, United States of America; k Department of Biomedical Engineering, Washington University in St Louis, St. Louis, MO 63110, United States of America; l Department of Neuroscience, Washington University School of Medicine, St. Louis, MO 63110, United States of America; m Department of Psychological and Brain Sciences, Washington University in St. Louis, MO 63110, United States of America; n Program in Occupational Therapy, Washington University School of Medicine, St. Louis, MO 63110, United States of America; o Department of Pediatrics, Washington University School of Medicine, St. Louis, MO 63110, United States of America; p Department of Neurology, New York University Langone Medical Center, New York, NY 10016, United States of America

## Abstract

Diffusion imaging aims to non-invasively characterize the anatomy and integrity of the brain’s white matter fibers. We evaluated the accuracy and reliability of commonly used diffusion imaging methods as a function of data quantity and analysis method, using both simulations and highly sampled individual-specific data (927–1442 diffusion weighted images [DWIs] per individual). Diffusion imaging methods that allow for crossing fibers (FSL’s BedpostX [BPX], DSI Studio’s Constant Solid Angle Q-Ball Imaging [CSA-QBI], MRtrix3’s Constrained Spherical Deconvolution [CSD]) estimated excess fibers when insufficient data were present and/or when the data did not match the model priors. To reduce such overfitting, we developed a novel Bayesian Multi-tensor Model-selection (BaMM) method and applied it to the popular ball-and-stick model used in BedpostX within the FSL software package. BaMM was robust to overfitting and showed high reliability and the relatively best crossing-fiber accuracy with increasing amounts of diffusion data. Thus, sufficient data and an overfitting resistant analysis method enhance precision diffusion imaging. For potential clinical applications of diffusion imaging, such as neurosurgical planning and deep brain stimulation (DBS), the quantities of data required to achieve diffusion imaging reliability are lower than those needed for functional MRI

## 1. Introduction

Brain function is critically dependent on white matter tracts for interlobe communication (Laughlin and Sejnowski, 2003). Studies of white matter connecting distant regions of the brain have greatly advanced our understanding of systems-level brain organization (Mori et al., 2005). Damage to white matter via dysmyelination, demyelination, stroke, or trauma, is a key component of many neurological disorders (Corbetta et al., 2015; Lassmann et al., 2007; Mac Donald et al., 2011; Rizzo et al., 2012).

Diffusion imaging is an MRI technique that provides information about water diffusion, which can in turn be used to probe white matter organization. Classic diffusion tensor imaging (DTI) entails acquisition of multiple diffusion weighted images (DWI), each of which is sensitized to water diffusion in a particular direction. At least six orthogonally oriented DWIs are required to estimate a single diffusion tensor representing the orientation of white matter fibers at a given location in the brain (Basser et al., 1994a, Basser et al., 1994b; Pierpaoli et al., 1996). Several shape and orientation characteristics may be extracted from the estimated diffusion tensor: fractional anisotropy (FA), radial diffusivity (RD), axial diffusivity (AD), mean diffusivity (MD), and orientation angles *θ* and *ϕ*. While a model describing a single tensor is theoretically adequate for simple fiber pathways, the single-tensor model does not adequately describe the complex geometry of multiple crossing fibers. More complex models potentially can account for multiple diffusion compartments and thus resolve crossing fibers (Behrens et al., 2003; Ferizi et al., 2014; Jbabdi et al., 2012; Jensen et al., 2005; Tournier et al., 2013, 2019; Tuch, 2004; Zhang et al., 2012). Microstructure models using specialized imaging sequences have attempted to further probe tissue characteristics even further (Fan et al., 2021; Palombo et al., 2020; Wang et al., 2014, 2015; Zong et al., 2021).

Early diffusion imaging studies acquired the minimum requirement of six orthogonal DWIs for computing a single diffusion tensor (Pierpaoli et al., 1996). With improvements in MRI hardware and software and the demand for more complex diffusion models, acquisition schemes have increased in complexity. Clinical diffusion imaging studies typically acquire 12–30 DWIs per patient while research studies typically acquire 30–60 DWIs per participant (Jones and Cercignani, 2010). Recent large sample studies such as the Human Connectome Project (HCP, (Van Essen et al., 2012)) and the Adolescent Brain Cognitive Development (ABCD, (Casey et al., 2018)) study, collected 297 and 103 DWIs per participant, respectively. Collecting even more data per individual, through repeated sampling has been informative for functional MRI (precision functional mapping [PFM]) (Braga and Buckner, 2017; Gordon et al., 2017; Laumann et al., 2015), revealing previously undetected individual variants in functional network architecture (Gordon et al., 2021; Gratton et al., 2018; Greene et al., 2020; Marek et al., 2018; Newbold et al., 2020; Sylvester et al., 2020; Zheng et al., 2021). By analogy, intensive acquisition of DWIs in individuals could be similarly fruitful in the study of structural brain connectivity. Prior studies have examined the reliability and accuracy of diffusion imaging using less than 60 diffusion directions (Hasan et al., 2001; Jones, 2004). Evaluated measures have included mean FA (Jones and Cercignani, 2010; Lebel et al., 2012; Ni et al., 2006), tractaveraged FA (Gordon et al., 2018; Luque Laguna et al., 2020), and capacity to resolve crossing-fiber models (Rokem et al., 2015; Tournier et al., 2013). Model reliability has also been evaluated using histological validation (Jones et al., 2020; Kuo et al., 2008; Panagiotaki et al., 2012; Schilling et al., 2018), in various tissue types (Alexander et al., 2001, 2019). However, it is unclear what degree of within-individual reliability may be achieved by collecting much larger quantities of DWI data.

Therefore, we acquired repeated DWI scans over multiple sessions. Three individuals were scanned on multiple days using the ABCD study sequence (Casey et al., 2018). This sequence includes 103 DWIs (96 diffusion encoding directions; 4 b-value shells; ~6.5 min). A total of 9 - 14 complete DWI datasets were acquired per individual. Differences in the head position across scans contributes additional variability in angular sampling for each subject. Thus, repeated scanning with the same sequence increases both angular sampling and SNR. These repeated sampling data were used to study how DWI data quantity and analysis methods impact reliability and accuracy. We pseudo-randomly sampled DWI encodings in a manner that maintained approximately constant angular coverage (see Methods; Figure S1), to systematically evaluate how reliability depends on angular sampling. Although earlier work has suggested that 30 spatially distributed DWIs could be sufficient to estimate a diffusion tensor (Jones, 2004), more complex models have not been similarly tested.

Four crossing-fiber estimation methods were compared: FSL’s BedpostX (BPX) (Behrens et al., 2003; Jbabdi et al., 2012; Sotiropoulos et al., 2016) uses the ball-and-sticks model and Automatic Relevance Determination (ARD) to select the number of fiber directions. The ball-and-sticks model was separately estimated using a novel Bayesian model selection developed in our laboratory which we term Bayesian Multi-Tensor Model selection (BaMM). The third and fourth crossing-fiber estimation methods tested here were DSI Studio’s Constant Solid Angle Q-ball Imaging (CSA-QBI; (Aganj et al., 2010; Tuch, 2004), and MRtrix3’s Constrained Spherical Deconvolution (CSD, (Tournier et al., 2013, 2019), two of the currently most widely used diffusion processing packages. As a control, we also tested two single-tensor estimation methods: linear least squares (LLS) and single-tensor Bayesian (STB) (Basser et al., 1994a, Basser et al., 1994b; Lee et al., 2010). These six diffusion modeling methods were selected as examples of differing approaches to diffusion imaging (Behrens et al., 2007; Jbabdi et al., 2012; Jensen et al., 2005; Tournier et al., 2013, 2019; Tuch, 2004; Wang et al., 2014, 2015; Zhang et al., 2012), but are not an exhaustive representation. Pertinent model estimation differences may be summarized as follows: BaMM and BPX both use a partial volume model assuming a variable number of radially symmetric fiber compartments. BaMM incorporates a model selection approach to estimate the number of fiber compartments. BPX uses automatic relevance determination (ARD) to down-weight unnecessary fiber compartments. CSA-QBI is a method derived from Q-ball numerical approximation of the water diffusion orientation distribution function (dODF) (Aganj et al., 2010; Callaghan et al., 1988; Tuch, 2004). The CSD method use a constrained spherical deconvolution to estimate the fiber orientation distribution (FOD) (Dell’Acqua and Tournier, 2019; Jeurissen et al., 2014). The accuracy and reliability of these methods was evaluated as a function of data quantity in both real and simulated data.

## 2. Methods

The organization of the present analyses is summarized in Table 1.

### 2.1. Voxelwise parameter estimation

We evaluated five parameter estimation methods: two methods (Bayesian Multi-Tensor Model-Selection [BaMM] and FSL’s BedpostX [BPX]) used the ball and sticks model (Behrens et al., 2003); the third crossing fiber method (Constant Solid Angle Q-Ball Imaging [QBI]) used spherical harmonics (Aganj et al., 2010); and two methods (Linear Least Squares [LLS] and Single Tensor Bayesian [STB]) used the classic single tensor model (Basser et al., 1994a).

#### 2.1.1. Bayesian multi-tensor model-selection (BaMM) modeling ball and sticks

We adapted a Bayesian model selection algorithm followed by parameter estimation of the winning model (modified from (Lee et al., 2010)). BaMM evaluated several competing models derived from the ball and sticks model (aka one ball vs. one ball and one stick; see Eq. (1)). Model selection and parameter estimation used a Markov-Chain Monte Carlo (MCMC), with Metropolis-Hastings sampling, and simulated annealing. The model selection penalty was scaled based on the input data size. Additional details on the implementation of this model are in the Supplemental Material.

#### 2.1.2. FSL’s BedpostX (BPX)

The ball and stick model, developed by FSL (Behrens et al., 2003), is an alternative to the single diffusion tensor model (Behrens et al., 2003; Jbabdi et al., 2012). BPX is a multi-compartment model, in which the first compartment models the diffusion of free water as isotropic (ball), and the rest of the *k* compartments model diffusion along several axial fiber directions with zero diffusion in the radial direction (sticks). The predicted diffusion signal is:

(1) $$\mu_{i}=S_{0}\left[(1-\Sigma_{k}f_{k})exp(-b_{i}d)+\Sigma_{k}f_{k}exp\left(-b_{i}d{\left(g_{i}^{T}x_{k}\right)}^{2}\right)\right]$$

where *i* indexes encoding direction and *k* indexes compartment. *S*_0_ is the signal with no diffusion weighting and *μ*_*i*_ is the signal with a diffusion gradient applied along the unit vector *g*_*i*_ with *b*-value *b*_*i*_ on diffusion signal *d*. The *f*_*k*_ are volume fractions for each fiber compartment. Each fiber compartment is modeled as a stick-like tensor oriented along *x*_*k*_. We employed FSL’s BedpostX 6.0.0 to evaluate BPX (Sotiropoulos et al., 2016). The Bayesian parameter estimation approach uses Automatic Relevance Determination (ARD) to down weight unnecessary fibers. BPX estimates angles *θ* and *ϕ* but not FA, MD, AD, or RD. Angles *θ* and *ϕ* are estimated for every direction (indeed by *k*). We ran BedpostX using the default settings unless noted otherwise: 2 fibers, weight = 1, and burn in = 1000.

#### 2.1.3. DSI studio’s constant solid angle Q-Ball imaging (CSA-QBI)

**Fig. 1.**
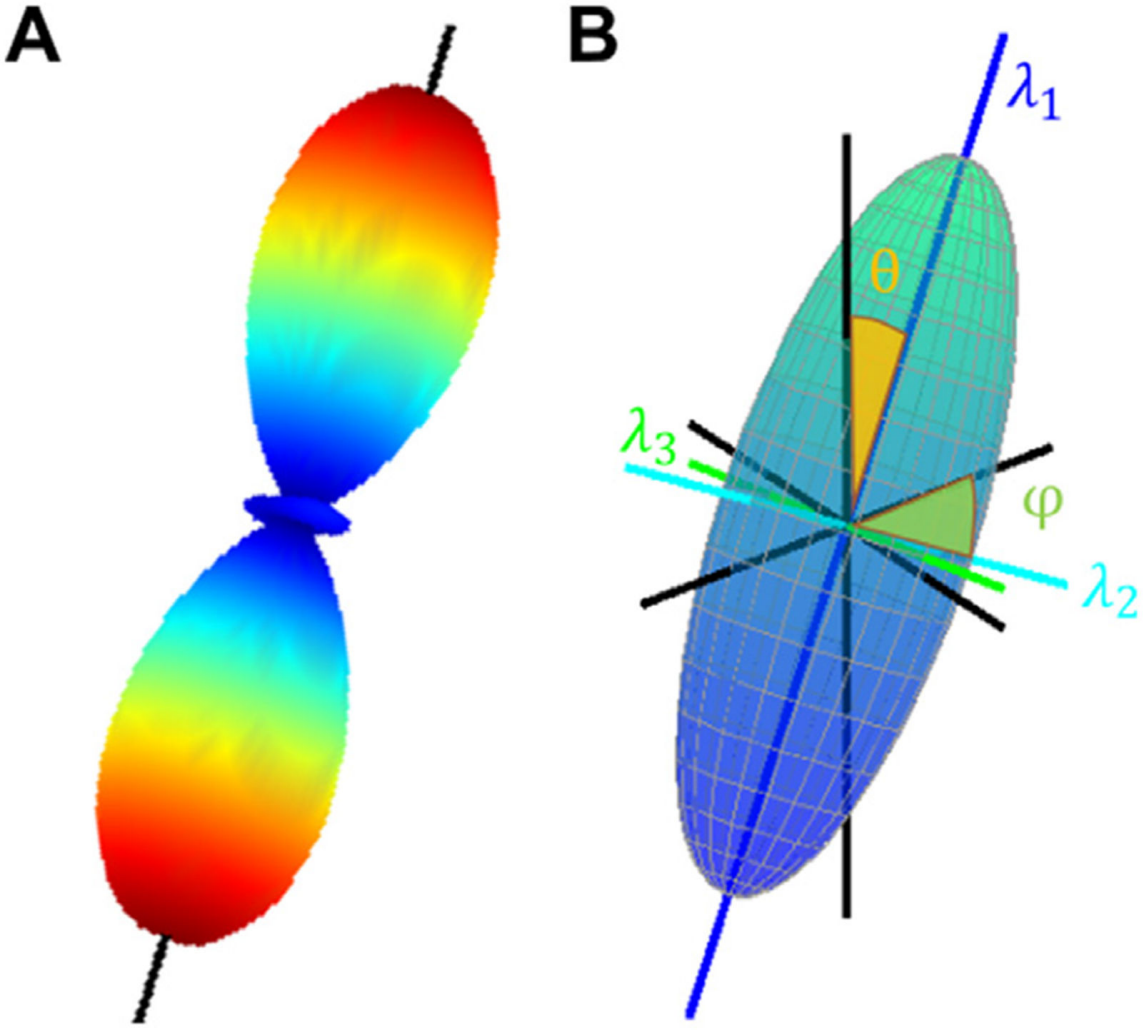
Estimated Tensor and Angles (A) Constant Solid Angle Q-Ball Imaging (QBI) reports fifteen spherical harmonic values, from which a 3D surface is estimated. The surface is colored by the orientation distribution function (ODF). The surface/ODF peaks are extracted (black line) and angles *φ* and *θ* estimated to match in B. (B) For Linear Least Squares (LLS) and Single Tensor Bayesian (STB), the tensor describing Brownian diffusion of water was calculated. Three eigenvalues are used to describe the tensor shape. From the largest eigenvector, two angles are estimated to describe the tensor orientation in 3D space. For Bayesian Multi-Tensor Model-Selection (BaMM) and FSL’s BedpostX (BPX), a stick corresponding to eigenvector-1 is estimated and its angles reported.

Q-ball imaging is a widely used reconstruction scheme available through DSI Studio that estimates the diffusion orientation distribution function (dODF) through a spherical tomographic inversion (Tuch, 2004). QBI was derived from q-space formalism (Callaghan et al., 1988) and uses a Funk-Radon transform to estimate the dODF. The original Q-ball imaging improved the dODF estimation by considering the constant solid angle (CSA-QBI; (Aganj et al., 2010)). CSA-QBI was downloaded from NITRC (nitrc.org) in 2020. For ease of comparison to the other methods (LLS, STB, BPX, BaMM, and CSD), we estimated the angle of the peaks given by the dODF surface generated by CSA-QBI (Fig. 1A). Peaks were selecting based on a normalized dODF probability greater than 0.3 and with a matching antipodal peak defined as two peaks having an absolute value dot product greater than 0.99.

#### 2.1.4. MRtrix3’s constrained spherical deconvolution (CSD)

Constrained spherical deconvolution (CSD) is currently one of the most cited reconstruction schemes available through MRtrix3 (Tournier et al., 2007, 2013, 2019). This method uses a constrained spherical deconvolution to estimate the fiber orientation distribution (FOD) and implements a regularized spherical deconvolution to deconvolve the signal with a single fiber response function. The following MRtrix3 functions with default settings were used unless otherwise noted: dwi2response with default tournier flag; dwi2fod with default csd flag; sh2peaks to extract XYZ coordinates of default top three peaks. For ease of comparison to the other methods (LLS, STB, BaMM, BPX, and CSA-QBI), the second and third peaks were included as a fiber direction if their magnitude was at least 10% of the maximal peak. The XYZ coordinates were then converted to spherical polar coordinates for consistency of presentation.

#### 2.1.5. Linear least squares (LLS)

The LLS method solves an overdetermined system of linear equations by single value decomposition (Basser et al., 1994a; Tristán-Vega et al., 2012). The solution yields a diffusion tensor *D*, which can be decom-posed into eigenvalues (*λ*_1_, *λ*_2_, *λ*_3_ Fig. 1B) and eigenvectors (*ν*_1_, *ν*_2_, *ν*_3_). Derived quantities from FSL’s ‘dtifit’ are fractional anisotropy (FA), radial diffusivity (RD), axial diffusivity (AD), mean diffusivity (MD). In addition, the orientation of the principal axis of diffusion can be characterized in terms of polar angles relative to the Z-axis (*θ*) and azimuthal rotation in the XY plane (*ϕ*, Fig. 1B (Behrens et al., 2003).

#### 2.1.6. Single tensor Bayesian estimation (STB)

We wanted to compare the LLS single tensor fit to a Bayesian estimation that used biological priors and had a non-negative constraint. The single tensor Bayesian method estimates the posterior probability of the set of parameters, *ω*_*i*_ = (*θ*, *ϕ*, *λ*_1_, *λ*_2_, *λ*_3_, *S*_0_) in voxel *j*, given the single tensor model *M* with relevant background information *I*:

(2) $$P(\omega_{i}|M_{j},I)\propto P(M_{j}|\omega_{i},I)P(\omega_{i}|I)$$

The background information *I* is given as several priors that reflect biological constraints: *λ*_1_, *λ*_2_, *λ*_3_ were limited to between 0 and 3 mm^2^/s, the biological range of diffusion in white matter. We assumed rotational symmetry; *λ*_2_, *λ*_3_ were set equal to each other, and *θ*, *ϕ* were limited to between 0 and *π* owing to the directional symmetry of the diffusion tensor. In STB, diffusion is modeled as a tensor, *M* (see Eq. (3) below). To estimate the model parameters, we used standard Monte Carlo Markov Chain methods (Lee et al., 2010).

### 2.2. Simulated data

Simulated data were generated using the Gaussian tensor model (Basser et al., 1994a, Basser et al., 1994b):

(3) $$\mu_{i}=S_{0}\left[exp\left(-b_{i}\cdot x_{i}Rx_{i}^{\prime }\right)\right],$$

where *S*_0_ is the signal with no diffusion weighting, *μ*_*i*_ is the signal with a diffusion gradient applied along the vector *x*_*i*_ with b-value *b*_*i*_, and *R* is the diffusion tensor. *S*_0_, was fixed at 1000; *b*_*i*_ and *x*_*i*_ matched twelve acquisitions of the ABCD sequence (Casey et al., 2018). Three cases were simulated:

#### 2.2.1. Single tensor

The first test case simulates highly organized white matter with a single principal direction, as in the mid-sagittal part of the corpus callosum. *R* was defined to have an anisotropy of 0.86, with angles *θ* and *ϕ* set to 1.8 and 2.8 radians, respectively. Gaussian noise was added independently to a real and imaginary channel which were combined as a magnitude to produce simulated data with Rician noise (Soares et al., 2013). We specified the signal to noise ratios (SNR) of 30, 50, 100, relative to the b0 to create three data sets with varying SNR.

#### 2.2.2. Two crossing tensors

The second test case simulates two highly organized, crossing white matter tracts. The simulations were generated as two highly anisotropic tensors, *R*_1_ and *R*_2_. A range of possibilities was explored by varying the SNR, tensor fraction, FA, and crossing angle of the tensors. Values were varied as follows: SNR = 30, 50, 100; tensor fractions of equal weighting (50%:50%) and unequal weighting (60%:40%, 70%:30%); FA = 0.6:0.6, 0.6:0.8, 0.8:0.8; crossing angle = 30°, 60°, 90°.

#### 2.2.3. Three crossing tensors

The final test case simulates three highly organized, crossing white matter tracts. The simulations were generated as three highly anisotropic tensors, *R*_1_, *R*_2_, and *R*_3_. A range of possibilities was explored by varying the SNR, tensor fraction, FA, and crossing angle of the tensors. Values were varied as follows: SNR = 30, 50, 100; tensor fraction equal weighting (33%:33%:33%) and unequal weighting (40%:34%:26%, 53%:34%:13%); FA = 0.6:0.6:0.6, 0.7:0.7:0.7, 0.8:0.8:0.8; crossing angle = 30°, 60°, 90°.

### 2.3. Repeatedly sampled individual-specific data

#### 2.3.1. Participants and study design

Three individuals who participated in a study of the effects of arm immobilization functional connectivity contributed data (Newbold et al., 2021, 2020). Participants (25yoF, 27yoM, 35yoM) were scanned daily for two weeks prior to an experimental intervention (unilateral arm casting). Imaging was performed at a consistent hour of the day to minimize diurnal effects. Data acquired during and after the casting period are not analyzed in this paper. Since Subject 1 (35yoM) did not have DWI data acquired prior to the experimental intervention, he was rescanned with the same sequence as the other subjects at later date. The Washington University School of Medicine Institutional Review Board provided experimental oversight. Participants provided informed consent for all aspects of the study and were paid for their participation.

#### 2.3.2. MR image acquisition

All MRI data were acquired on a Siemens 3T Prisma using a 64-channel head coil, structural MRI was acquired at each scanning session and included T1-weighted images (Gradient echo, 3D MP-RAGE, sagittal, 300 slices, 0.8 mm isotropic resolution, TR/TE=2400/2.22 ms, TI=1000 ms, flip angle = 8°), and T2-weighted images (Spin echo, 3D T2-SPC, sagittal, 300 slices, 0.8 mm isotropic resolution, TR/TE=3200/563 ms) (Newbold et al., 2021, 2020).

Daily scans used the ABCD diffusion sequence, a single-shot echo planar diffusion-weighted MRI with the following sequence parameters: 75 contiguous axial slices, 2 mm isotropic resolution, TR/TE 3500/83 ms, four shells (b-values 250, 500, 1000, and 1500s/mm^2^). This sequence includes 103 vol and 96 encoding directions (Casey et al., 2018). Acquisition time per scan was 6.5 min, and a single acquisition was collected on each scan day. Total DWI scans (distributed across scanning sessions) for the three subjects were 9, 12, and 14, resulting in a total of 864, 1152, and 1440 total encoding directions, respectively. Two field maps (AP and PA) were acquired with the same settings as the diffusion weighted data for subsequent processing.

Subject 1 was also scanned using a custom set of diffusion gradients, with all other ABCD sequence parameters kept the same. This scan is referred to as the single session high angular resolution (SS-HAR) scan. The sequence parameters were as follows: 75 contiguous axial slices, 2 mm isotropic resolution, TR/TE 3500/83 ms, four shells (b-values 250, 500, 1000, and 1500s/mm^2^), 1020 vol with 960 unique encoding directions. Acquisition time was 1 hr. Two b0 acquisitions with reverse phase-encoding direction (AP and PA) were acquired with the same settings as the diffusion weighted data for estimation of the field map.

#### 2.3.3. DWI processing

We applied FSL’s Eddy current correction and top-up (Andersson and Sotiropoulos, 2016; Smith et al., 2004) to each DWI acquisition. During eddy correction, FSL calculated total movement of each DWI relative to the first volume. We excluded volumes with framewise displacement greater than 0.5 mm (Baum et al., 2018). The mean and standard deviation of displacement in millimeters relative to the prior volume for each subject were: 0.24 and 0.13 for Subject 1; 0.29 and 0.19 for Subject 2; and 0.38 and 0.23 for Subject 3. Each DWI acquisition was affine registered to the participant’s structural T1 data, and gradient vectors were transformed accordingly before concatenating all diffusion data within an individual. Diffusion tensor maps were computed using FSL’s tool DTIFIT (Jenkinson et al., 2012). FSL’s eddy correction also generates rotation corrected b-vectors used in the subsequent processing (STB, BaMM, BPX, CSA-QBI, CSD).

#### 2.3.4. Creation of reliability curves using permutation resampling

**Fig. 2.**
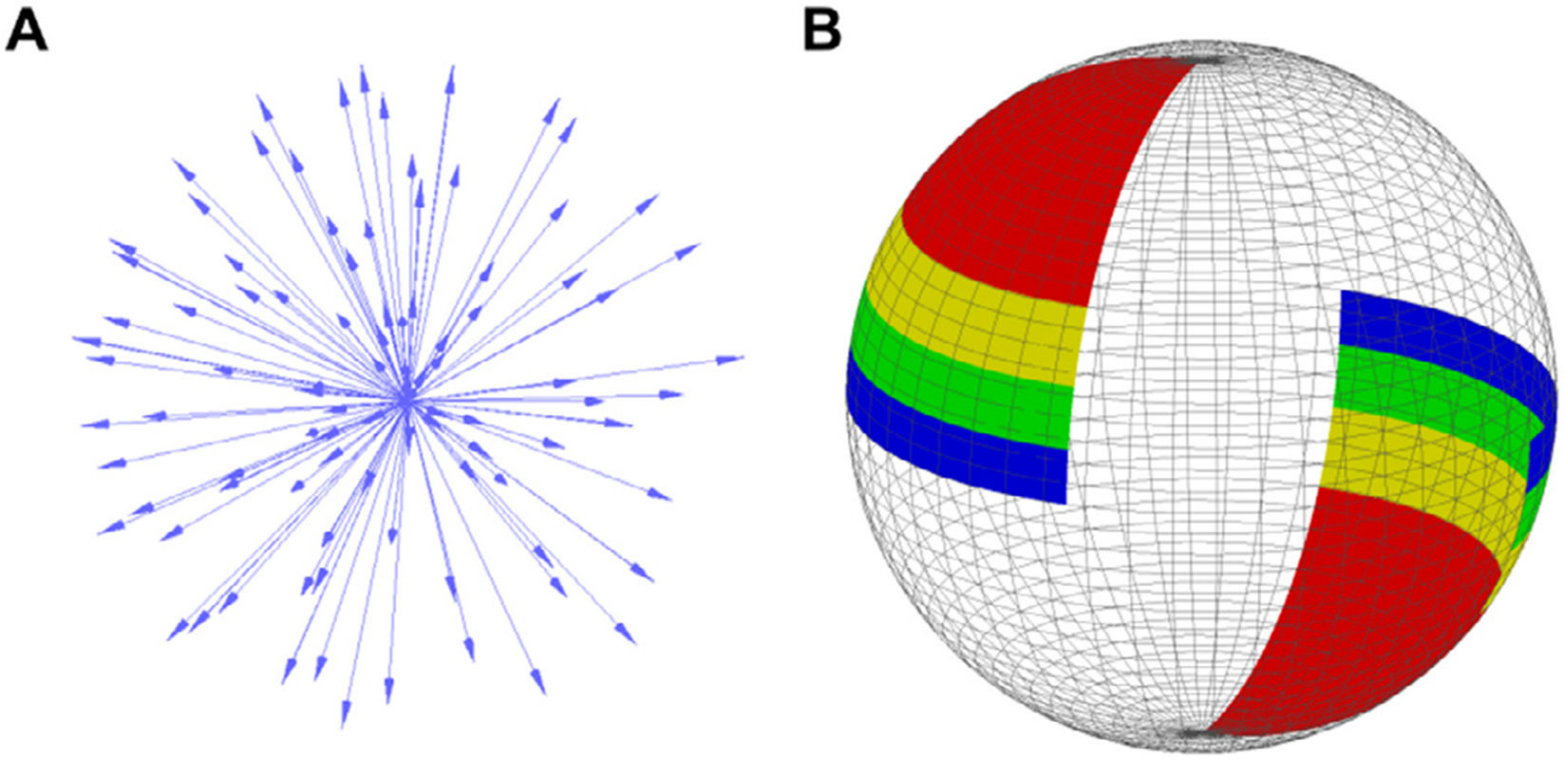
B-Vector selection Subjects were scanned every day for two weeks, with 96 unique B-vector directions acquired each scan. A) All 1152 B-Vectors from the daily scans plotted on a single sphere. B) B-Vectors were subdivided by their position on the sphere into 16 groups of equal surface area, 4 of which are shown. Encodings were pseudo-randomly selected from the 16 groups to obtain approximately uniform angular sampling over the sphere.

Model estimation with permutation subsampling was used to quantitatively estimate modeled parameter variability. This approach was used for both simulated data and real human data. All available DWI volumes acquired across 9–14 scanning sessions were concatenated. Subsamples covered the shell surface approximately evenly (Fig. 2A). Solid angle sectors were defined by dividing the shell into sixteen bidirectional groups (Fig. 2B). The XY-plane was divided into four quadrants and polar angle (*θ*) was divided into four intervals equating arclength. For each permutation, we pseudo-randomly sampled DWI encodings in a manner that maintained approximately constant angular coverage (Figure S1).

For all exemplar parameter estimation methods (BaMM, BPX, QBI, LLS, STB), we compared the estimation of relevant modeled diffusion parameters ({*θ*, *ϕ*} for all models, {FA, AD, RD, MD} for relevant subset) over the range *N* = 10:1000, in approximately logarithmically spaced increments. Specifically, we used a step size of 10 for values of N below or equal to 200, then a step size of 20 for values of N between 200 and 300, a step size of 40 for values of N between 300 and 500, and a step size of 50 for values between 500 and 1000. DWI volumes were quasi-randomly selected according to the above-described scheme. These steps were repeated over 1000 permutations at each subsampling size. For single tensor shape diffusion parameters {FA, AD, RD, MD}, the parameter estimate variability was defined as

(4) $${\overset{‒}{e}}_{N}=\langle {\left(x_{i}-\chi_{T}\right)}^{2}\rangle^{1∕2}\;∕\;\chi_{T}$$

Where *x*_*i*_ represents a parameter estimate {FA, AD, RD, MD} obtained from a single permutation; *χ*_*T*_ is the ground truth as specified when generating the tensors in the simulations, or the estimated value obtained when using all available human neuroimaging data; the bracket denotes mean over permutations. ${\overset{‒}{e}}_{N}$ was plotted as a function of sample size (*N*), creating reliability curves for each parameter.

Since diffusion is estimated as a bipolar tensor that is symmetric around the origin, the error estimation for angles *θ*, *ϕ* was modified accordingly to account for modulus pi.

#### 2.3.5. Mean error threshold whole brain maps

To generate a voxel-wise heatmap visualizing the threshold sample size *N* needed to reach a mean error less than 5% for each voxel, we conducted the permutation testing described above on every voxel of the brain using the LLS method. The mean error was calculated for each voxel at each value of *N*. A heatmap was created for each diffusion metric, such that voxels are colored by the number of measurements needed to reach a mean error < 5%.

## 3. Results

### 3.1. Single tensor simulations: BaMM and CSD detect a single fiber more accurately than BPX or CSA-QBI

**Fig. 3.**
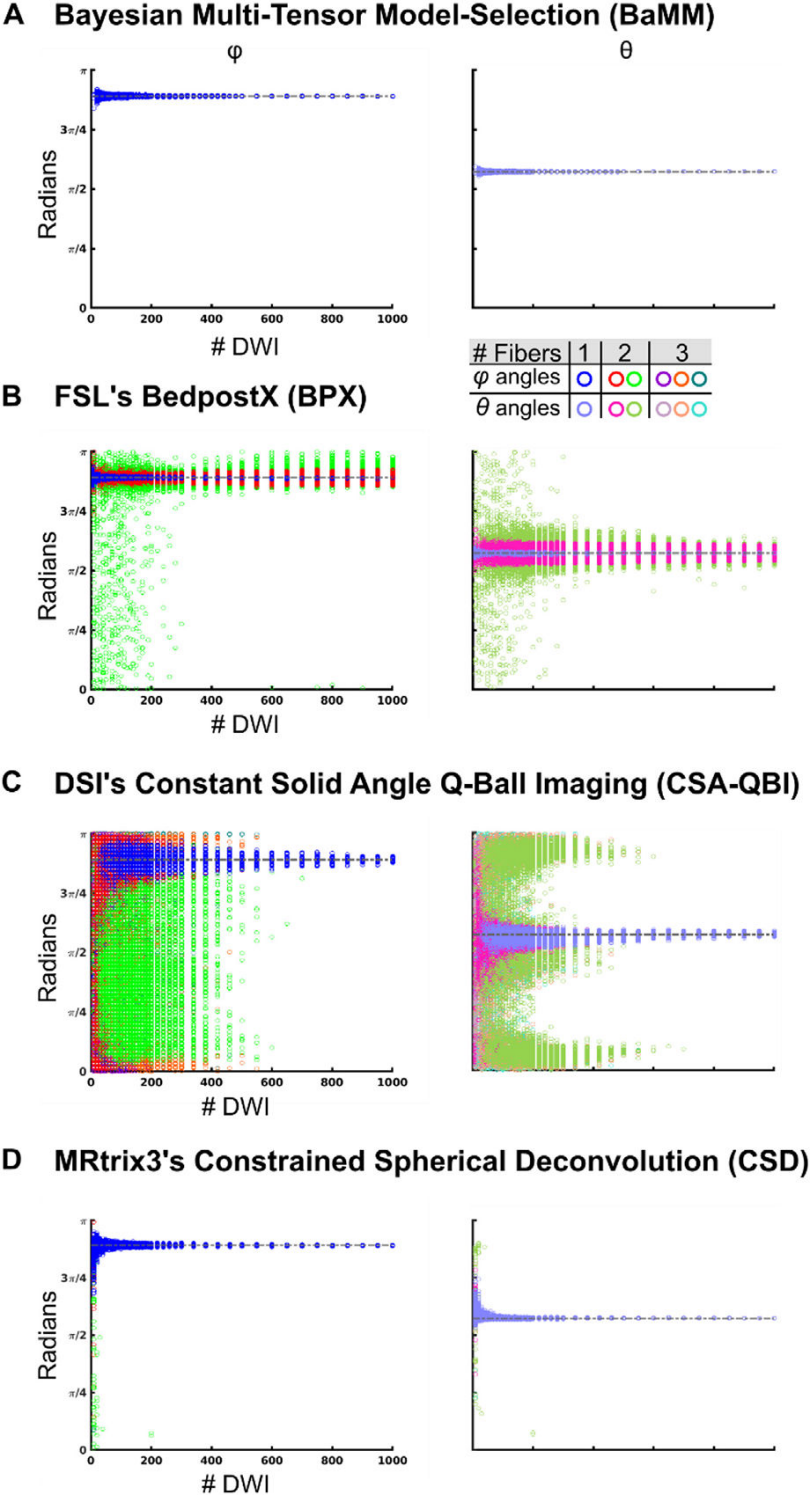
Accuracy of Diffusion Measures: Simulated Single Tensor (A) *φ/θ* angle estimations by Bayesian Multi-tensor Model-selection (BaMM). Open circles represent the results obtained by repeated permutation sampling. Same color legend for all data panels. Permutations that resulted in a single fiber direction are plotted in blue/sky blue (*φ/θ*). Permutations that resulted in two fibers are plotted in red/pink (*φ/θ*) and green/olive (*φ/θ*). Permutations that resulted in three fibers are plotted in purple/lilac (*φ/θ*), orange/salmon (*φ/θ*), and teal/cyan (*φ/θ*). (B) FSL’s BedpostX (BPX). (C) Constant Solid Angle Q-Ball Imaging (CSA-QBI). (D) Constrained Spherical Deconvolution (CSD).

Some regions of the brain, such as the corpus callosum, have a single dominant fiber direction. Thus, we first tested diffusion imaging methods with simulated single tensor fiber data. To evaluate the accuracy and reliability of diffusion metrics, we used permutation subsampling of the simulated diffusion data to estimate parameter variability for all crossing fiber models (BaMM [Bayesian Multi-tensor Model-selection], BPX [BedpostX], CSA-QBI [Constant Solid Angle Q-Ball Imaging], CSD [Constrained Spherical Deconvolution]) and single tensor methods (LLS [Linear Least Squares], STB [Single Tensor Bayesian]). We plotted the estimated radian value of the fiber (or tensor) angles (*ϕ*, *θ*) to highlight the number of fibers estimated at each DWI sample size. Open circles represent the results of individual permutations and are colored according to the number of fibers estimated (Fig. 3).

Multiple SNR values were tested to track the effect of SNR on reliability and accuracy. The forward-modeled parameter space for simulated single tensors was: SNR 50 (Fig. 3), 30, and 100 (Figure S2 for BaMM, BPX, CSA-QBI and CSD, and S3 for LLS and STB). Initially, default settings were used for all modeling schemes: BaMM, CSA-QBI and CSD up to three fibers; BPX two fibers (for same analyses using BPX with other settings see Figure S4).

BaMM accurately estimated the orientation of the single forward modeled principal eigenvector, even with limited quantities of data (> 20 DWI samples; blue/sky blue symbols Fig. 3A, S2).

BPX generally falsely estimated two fibers (69% of permutations at DWI = 10, at least 90% of permutations at DWI > 120), even when given large quantities of data (Fig. 3B, S2). At DWI < 200, the angle of the second fiber was broadly distributed over the interval 0 to *π* (green/olive symbols Fig. 3B). At DWI > 400, BPX continued to estimate two fibers separated by a small angle, the mean of which closely approximated the single modeled principal eigenvector. When the max number of fibers was increased to 3 (default 2), BPX falsely estimated three fibers in the majority of permutations (39% of permutations at DWI = 10, linearly increasing to 88% of permutations at DWI = 1000; Figure S4).

QBI estimated one, two or three fibers given different numbers of DWI (Fig. 3C, S2). At < 90 DWI, QBI was most likely to estimate three fibers that were broadly distributed over the interval 0 to *π*, and also frequently estimated one or two; at 10 DWI, 90% of permutations estimated three fibers, 10% estimated two. By 80 DWI, 47%, 42%, and 11% of permutations estimated three, two, and one fiber, respectively. Unlike BPX, QBI consistently and accurately estimated a single fiber at higher DWI quantities (300 DWI: 12%, 31%, and 57% of permutations estimated three, two, and one fiber, respectively). Over 90% of QBI permutations estimated a single fiber at > 460 DWI.

CSD also accurately estimated the orientation of the single forward modeled principal eigenvector with limited quantities of data (> 20 DWI samples; Fig. 3D).

Mean measurement error was calculated relative to the forward-modeled angle or shape metric, to quantify the accuracy of each method as a function of the number of diffusion measurements (Figure S2-3, Eq. (4)). For BaMM and CSD, error linearly decreased with increasing subsampling size. In contrast, for BPX a linear decrease of error with increasing subsampling measurements was detected only for the secondary fiber but not the primary fiber. QBI’s error decreased with increasing subsampling sizes only for the primary fiber, while the second and third fiber had very high errors.

We also evaluated the accuracy of the single tensor methods LLS and STB on simulated single tensor data. As expected and similar to BaMM and CSD, LLS and STB estimation of FA, AD, RD, MD, and angles *ϕ* and *θ* improved with increasing number of diffusion measurements (Figure S3).

### 3.2. Two tensor simulations: BaMM is robust against overfitting

**Fig. 4.**
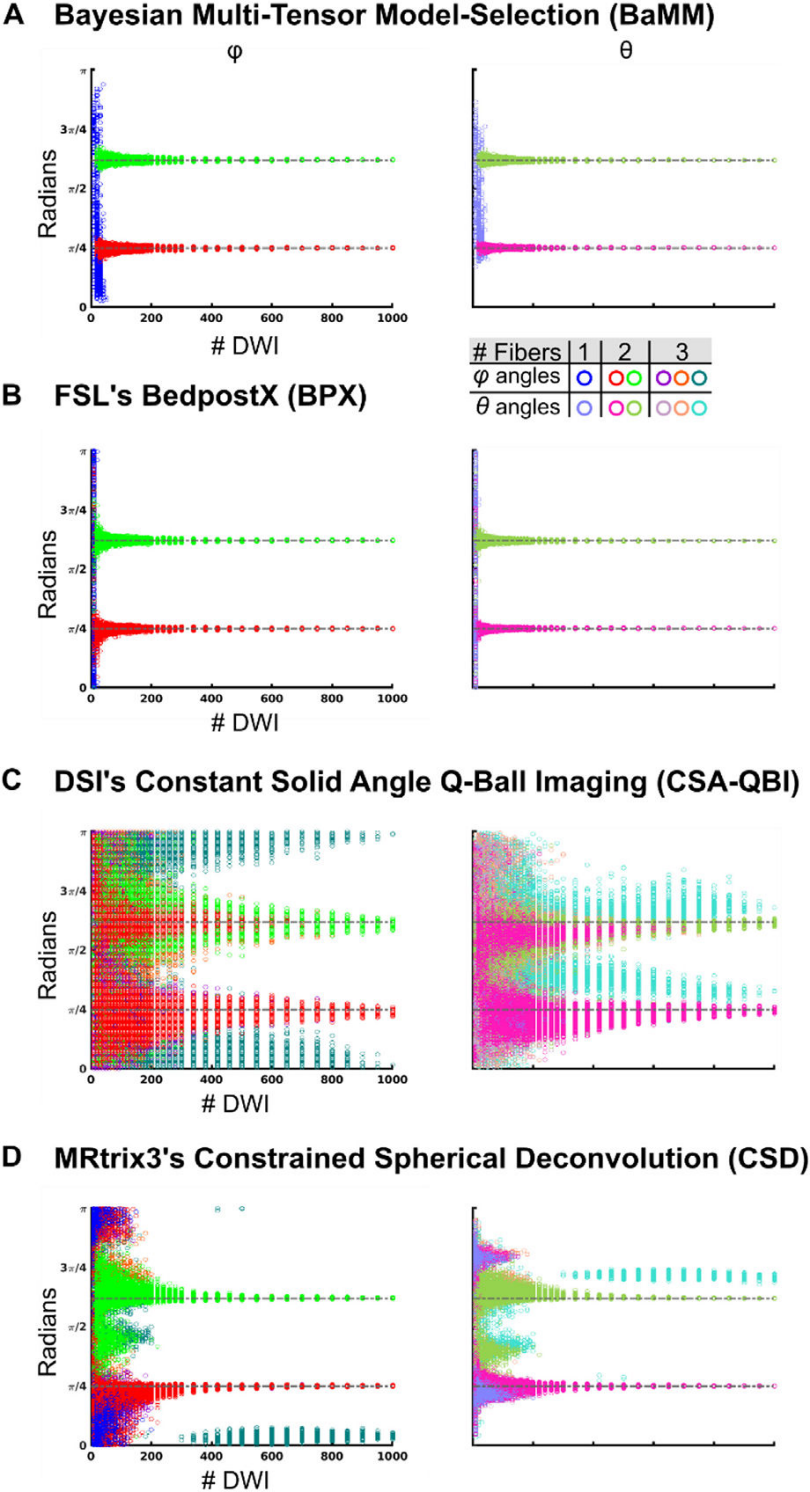
Accuracy of diffusion measures: simulated two crossing tensors The tensors were oriented such that they were perpendicular to each other. The first tensor had larger weighting equal to 60% of the signal. Rician noise was added for an SNR = 50. (A) *φ/θ* angle estimations by Bayesian Multi-tensor Model-selection (BaMM). Open circles represent the results obtained by repeated permutation sampling. Same color legend for all data panels. Permutations that resulted in a single fiber direction are plotted in blue/sky blue (*φ/θ*). Permutations that resulted in two fibers are plotted in red/pink (*φ/θ*) and green/olive (*φ/θ*). Permutations that resulted in three fibers are plotted in purple/lilac (*φ/θ*), orange/salmon (*φ/θ*), and teal/cyan (*φ/θ*). (B) FSL’s BedpostX (BPX). (C) Constant Solid Angle Q-Ball Imaging (CSA-QBI). (D) Constrained Spherical Deconvolution (CSD).

Next, we simulated two crossing fibers, as within the crossing of the superior longitudinal and uncinate fasciculi (Figs. 4, S5-8). We explored the following forward-modeled parameter space: fiber crossing angle (30°, 60°, 90°), relative weight of fiber compartments (50/50, 60/40, 70/30), SNR (30, 50, 100), and FA of tensors (0.6/0.6, 0.6/0.8, 0.8/0.8). The parameter space was chosen to explore fiber orientation, relative size of fiber compartments, SNR, and the respective FA of the fiber compartments. Fig. 4 shows the results of 90° crossing angle, 60/40 relative weight, SNR 50, and FA of 0.8/0.8. Results corresponding to the full parameter space are reported in the Supplemental Figures (Figures S5-8, BaMM, BPX, CSA-QBI, and CSD respectively). Results were consistent across the parameter space, with slight variations in the subsampling size needed to reach specific error thresholds. Single tensor models (LLS and STB) estimated the two crossing fibers as a weighted average and the single tensor’s principal eigenvector, which reflects the inaccurate shape assumption (Figure S9). Again, default settings were used for all modeling schemes: BaMM, CSA-QBI, and CSD up to three fibers; BPX two fibers (for same analyses of BPX using max 3-fiber settings see Figure S10).

BaMM consistently and correctly estimated two fibers for > 30 DWI (red/pink and green/olive symbols Fig. 4A, full parameter space in Figure S5).

BPX estimated two fibers at all but the smallest subsampling size (DWI = 10; Fig. 4B, S6) when using default settings. When we increased BPX’s maximum allowable number of fibers to 3 (Figure S9), BPX frequently estimated three fibers for all DWI subsamplings with consistently increased angular error.

For simulated two tensor data, similar to single tensor data, QBI also incorrectly estimated three fibers at DWI < 150 (Fig. 4C, S7). Even though two fiber directions were most commonly found at higher sampling density, some permutations still demonstrated three fiber directions at all subsampling sizes (62% at 100 DWI, 44% at 300 DWI, 33% at 500 DWI, 14% at 800 DWI).

CSD most frequently estimated two fibers for all sample sizes. With insufficient data (< 50 DWI) some permutations estimated one or three fiber directions (at 40 DWI, 9% estimated 1 direction and 9% estimated 3 directions). With larger data quantity (> 460 DWI), at least 10% of permutations estimated a third fiber direction.

### 3.3. Three tensor simulations: accurate estimates achieved with fewest DWIs using BaMM

**Fig. 5.**
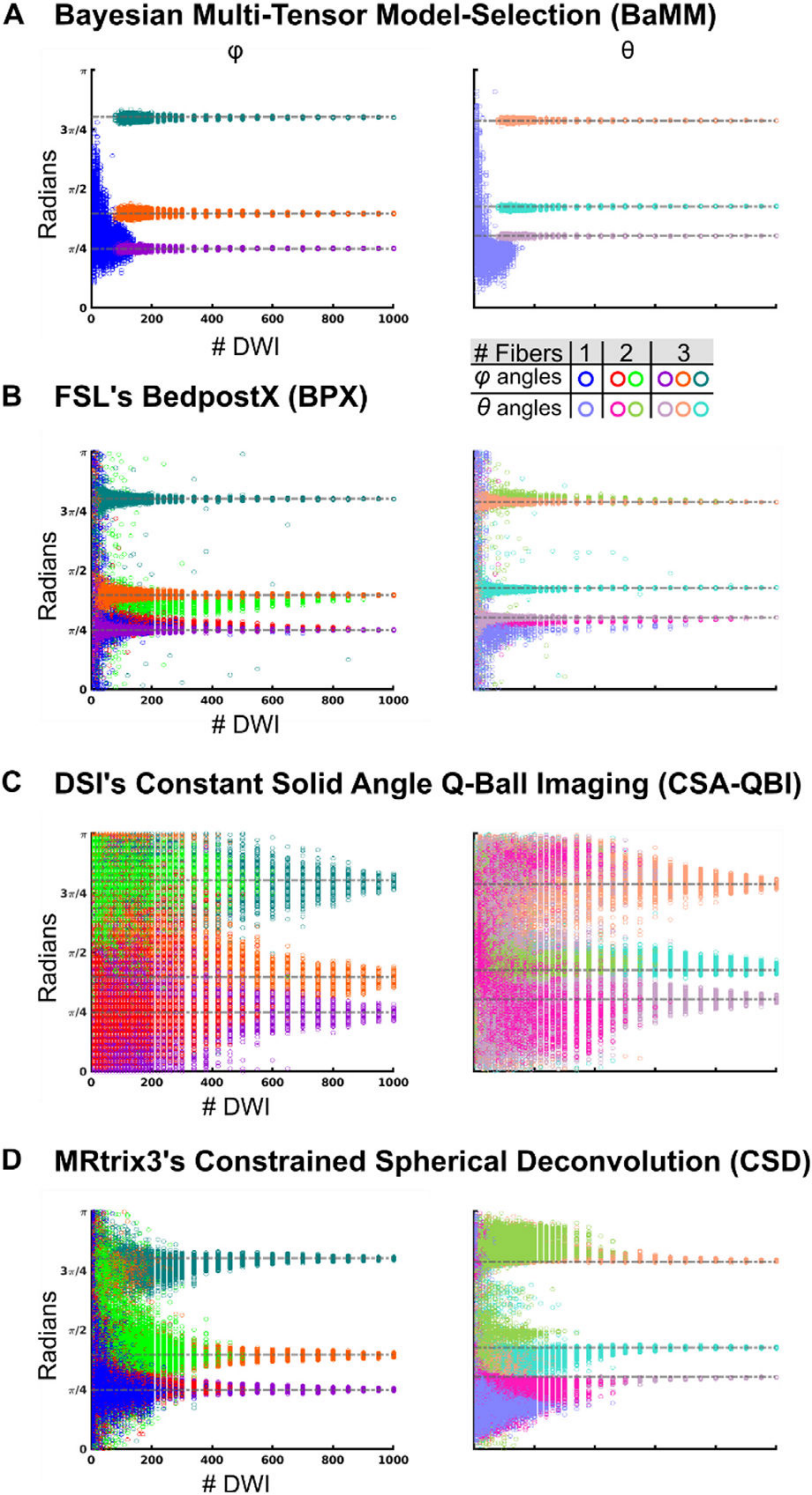
Accuracy of diffusion measures in simulated three crossing tensor data The tensors were oriented such that they were perpendicular to each other. The tensors had weighting equal to 40%, 34%, and 26% of the signal. Rician noise was added for an SNR = 50. (A) *φ/θ* angle estimations by Bayesian Multi-tensor Model-selection (BaMM). Open circles represent the results obtained by repeated permutation sampling. Same color legend for all data panels. Permutations that resulted in a single fiber direction are plotted in blue/sky blue (*φ/θ*). Permutations that resulted in two fibers are plotted in red/pink (*φ/θ*) and green/olive (*φ/θ*). Permutations that resulted in three fibers are plotted in purple/lilac (*φ/θ*), orange/salmon (*φ/θ*), and teal/cyan (*φ/θ*). (B) FSL’s BedpostX (BPX). (C) Constant Solid Angle Q-Ball Imaging (CSA-QBI). (D) Constrained Spherical Deconvolution (CSD).

The final simulation was of three crossing tensors, as in crowded areas of deep white matter, where the thalamic radiation, longitudinal tracts, and commissural tracts all cross. We explored the following forward-modeled parameter space: fiber crossing angle (30°, 60°, 90°), relative weight of fiber compartments (33/33/33, 26/34/40, 13/34/53), SNR (30, 50, 100), and FA of tensors (0.6/0.6/0.6, 0.7/0.7/0.7, 0.8/0.8/0.8). Fig. 5 shows the results of 90° crossing angle, 40/34/26 relative weight, SNR 50, and FA of 0.8/0.8/0.8. Results corresponding to the full parameter space are reported in the Supplement (Figures S11-14, BaMM, BPX, CSA-QBI, and CSD respectively). Results were consistent across most of the parameter space. All methods showed the lowest accuracy for the narrowest crossing fiber angle (30°).

BaMM consistently estimated three fiber compartments with sufficient data (DWI > 200, Fig. 5A, S11). We increased BPX’s max possible fibers to 3 to match the simulated data, and then BPX estimated three fiber compartments at all subsampling sizes (Fig. 5B, S12). As with prior modeling, BaMM and BPX correctly determined there were three fiber compartments and accurately estimated *ϕ* and *θ* for the three fibers with increasing sampling density.

QBI most frequently estimated three fiber directions at all sampling densities, yet often estimated one or two fibers < 500 DWI (Fig. 5C, S13).

For the base simulation set (90° crossing angle, 40/34/26 relative weight, SNR 50, and FA of 0.8/0.8/0.8), CSD consistently estimated three fiber directions with > 200 DWI and demonstrated increasing accuracy with increasing sampling density (Fig. 5D). The number of estimated fiber directions was less consistent for CSD than the other methods evaluated here, with varying number of fiber directions given slight changes in the simulation parameter set (Figure S14).

For the entire parameter space of the three tensor simulations, BaMM and BPX log errors decreased almost linearly with an increase in the number of diffusion measurements (Figure S11-12). QBI (Figures S13) approached the expected relationship between log error and sample size once three fiber directions were consistently estimated (> 500 DWI), yet still had higher error than all other methods at the largest subsampling sizes. CSD generally showed a linear relationship between log error and sample size when three fiber directions were estimated (Figure S14), but accuracy varied when fewer fiber directions were estimated.

### 3.4. Whole brain reliability mapping reveals very high data requirements in gray matter

**Fig. 6.**
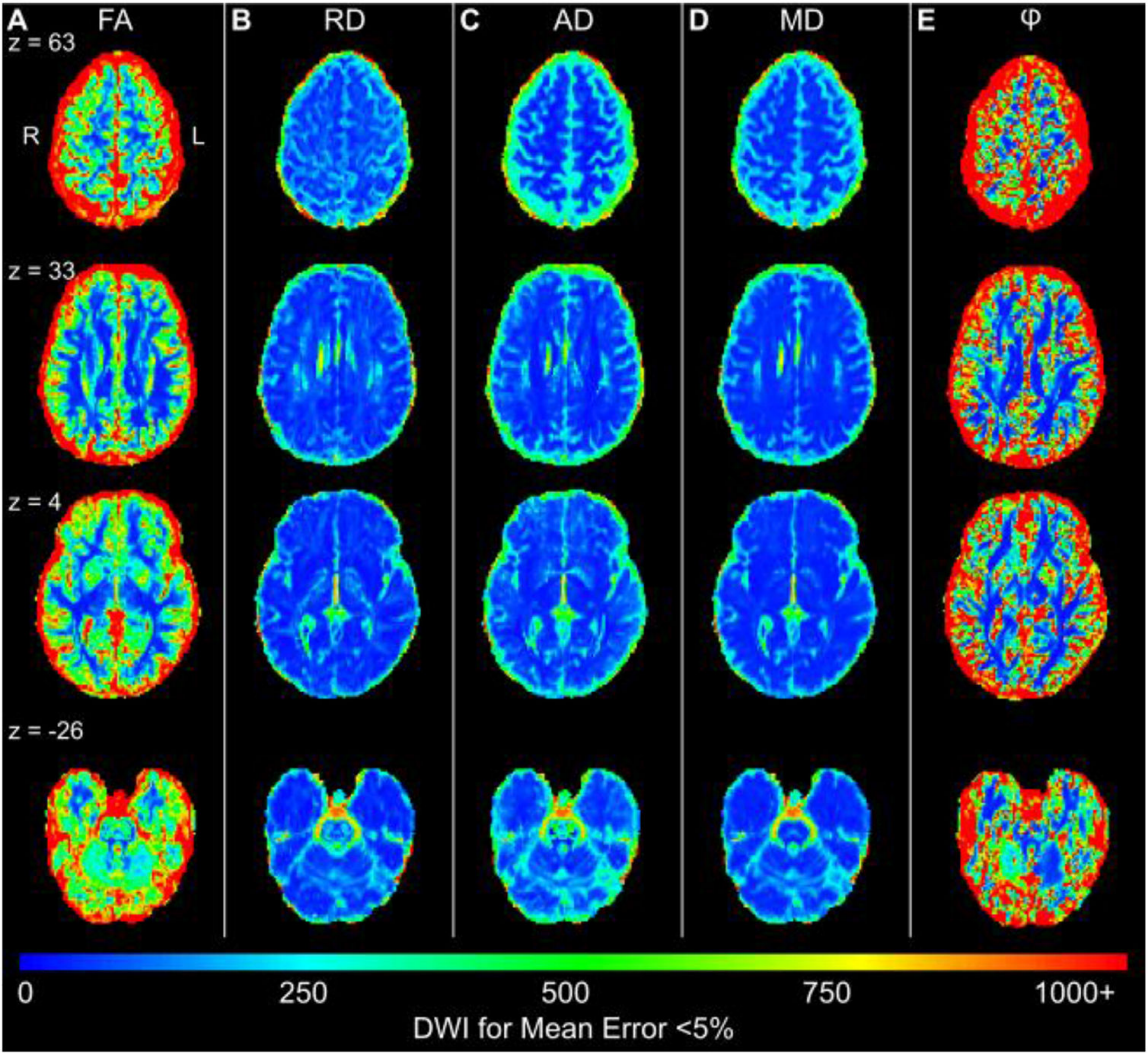
Whole-brain DTI reliability map (linear least squares) for mean error < 5% (Subject 2) (A) The color scale shows the number of DWI measurements needed to achieve a voxel-wise error less than 5% in FA. Error is calculated relative to the mean FA found using the entire sample. Results for (B) RD, (C) AD, (D) MD, and (E) angle *φ* are shown. Subjects 1 and 3 are shown in Figure S15, all subjects shown in S16.

To test reliability of diffusion metrics in human data (highly sampled, three participants), we used permutation subsampling of all available data to estimate whole-brain parameter variability (FA, RD, AD, MD, *ϕ*, *θ*) using the Linear Least Squares (LLS) method. LLS was used because none of the other methods were computationally tractable for whole brain analyses of this type, and whole brain reliability maps were desired to help identify anatomically defined regions of interest (ROIs). Fig. 6 shows the number of DWIs required to reach a mean error (RMSE) < 5% at each voxel in Subject 2 (Subject 1 and 3 shown in Figure S15). Subject 2 was chosen as the exemplar because they had the relatively best LLS reliability, (single slice inter-subject comparison in Figure S16). Error is now reported as the deviation from the mean when using the full sample ( Eq. (4)) rather than relative to the ground truth as in the prior simulations. AD, RD, and MD had less measurement error than FA and the angles *ϕ* and *θ* across most of the brain. In parts of the corpus callosum, only 20 DWIs were required for an FA RMSE < 5%. For most deep white matter voxels (e.g., corticospinal tracts, frontal crossing tracts), about 100 DWI samples were sufficient for an FA RMSE < 5%. In comparison, gray matter voxels required 300–500 measurements to reach an FA RMSE < 5%.

### 3.5. Corpus callosum: only BaMM estimates single fiber < 600 DWIs

**Fig. 7.**
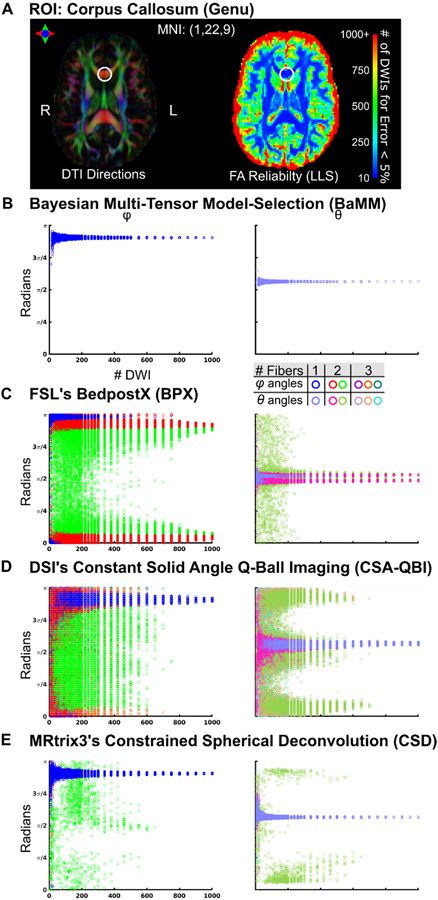
Reliability of diffusion measures in the genu of the corpus callosum (subject 2) (A) The locus of the analyzed voxel (MNI: 1, 22, 9) is marked with a circle. Linear Least Squares (LLS) FA reliability map as in Fig. 6A. (B) *φ/θ* angle estimations by Bayesian Multi-tensor Model-selection (BaMM). (C) FSL’s BedpostX (BPX). (D) Constant Solid Angle Q-Ball Imaging (CSA-QBI). (E) Constrained Spherical Deconvolution (CSD). Subject 1 and 3 in Figures S18-19, respectively.

To examine individual-specific diffusion metric reliability with highly sampled data, across methods, several ROIs were selected based on the whole-brain, voxel-wise LLS reliability maps (Fig. 6 and S15-16) and prior anatomical knowledge. Fig. 7 shows diffusion estimates in a voxel of the corpus callosum exemplifying highly anisotropic diffusion (Fig. 7A; MNI: −1, 22, 9; Subject 2). This ROI in Subject 2 with BPX max 3 fibers is shown in Figure S17. Permutation results for Subject 1 and 3 are in Figures S18-19, respectively. This corpus callosum ROI was chosen because it is strongly expected to contain only a single white matter fiber direction. Reliability curves for all methods and subjects (including LLS and STB) are shown in Figure S20. As in the simulated single tensor data, these single tensor estimation methods showed low error rates (now reflecting reliability rather than accuracy), even for low DWI numbers.

BaMM estimated only a single fiber in the corpus callosum (Fig. 7B), regardless of the number of DWIs in the subsample, with angles *ϕ* and *θ* closely matching the results observed with single tensor methods (see Fig. 3, S2-3).

In contrast, BPX consistently estimated two fibers in the corpus callosum across all numbers of DWIs for the default setting of two fibers max (Fig. 7C). Figure S17 shows that when BPX’s max fiber number was increased to three it started to estimate three fibers in the corpus callosum for higher numbers of DWIs. The BPX principal fiber (red/pink) generally matched the orientation obtained with BaMM, CSA-QBI, and CSD (Fig. 7). At low sampling density, the angle of the second fiber estimated by BPX was broadly distributed over the interval 0 to *π* (green/olive). For DWI counts > 400, BPX continued to estimate two fibers, the average of which matched the orientation found by the other methods.

Similar to the simulated data, CSA-QBI estimated three fibers for subsamples with ⟨ 200 DWI, two fibers < 400 DWI, and a single fiber for ⟩ 400 DWI (Fig. 7D). For subsamples with ⟨ 200 DWI, CSA-QBI was most likely to estimate three fibers that were broadly distributed over the interval 0 to *π*, and also frequently estimated one or two fibers. Unlike BPX, CSA-QBI consistently and accurately estimated a single fiber for ⟩ 400 DWI. The existing anatomical priors about the corpus callosum would suggest a single primary diffusion direction, matching BaMM’s results at all subsampling sizes and CSA-QBI’s with ~1000 DWIs.

CSD predominantly estimated a single fiber direction across all subsample sizes (Fig. 7E). A subset of permutations estimated a second fiber direction, and the proportion of permutations with a second fiber direction decreased with increasing sample size (<10% with > 20 DWI, ⟨5% with ⟩ 300 DWI).

### 3.6. Left frontal white matter: BPX with two fiber default setting reliable with fewest DWIs

**Fig. 8.**
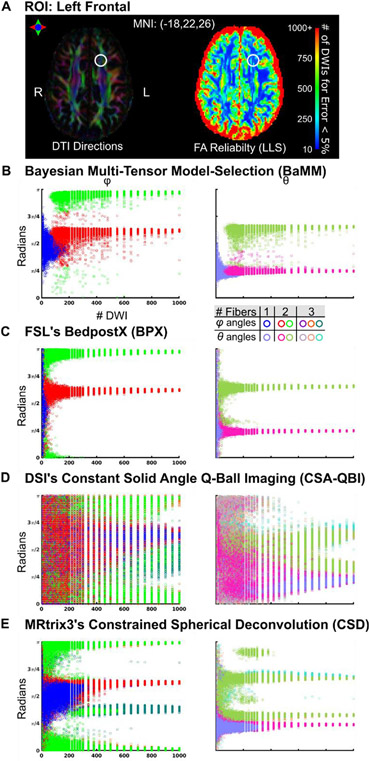
Reliability of diffusion measures in left frontal region (Subject 2) (A) The locus of the analyzed voxel (MNI: 18, 22, 26) is marked with a circle. Linear Least Squares (LLS) FA reliability map as in Fig. 6A (B) *φ/θ* angle estimations by Bayesian Multi-tensor Model-selection (BaMM). (C) FSL’s BedpostX (BPX). (D) Constant Solid Angle Q-Ball Imaging (CSA-QBI). (E) Constrained Spherical Deconvolution (CSD). Subject 1 and 3 in Figures S22-23, respectively.

We next selected a voxel in the left frontal lobe (MNI −18, 22, 26) where the superior longitudinal fasciculus and the uncinate fasciculus cross (Fig. 8A). This voxel was chosen to be > 10 mm from any gray matter voxel in all three subjects. This ROI in Subject 2 with BPX max 3 fibers is shown in Figure S21, and Subjects 1 and 3 in Figures S22-23, respectively. Reliability curves for all methods and subjects are shown in Figure S24. The single tensor models are inadequate to describe the full microstructural complexity, and increased error can be observed in Figure S20 vs. Figure S24.

Fig. 8B-E contrasts the angle measurement reliability of the crossing fibers models (BaMM, BPX, CSA-QBI, and CSD). For very low numbers of DWI per subsample (< 50), BaMM identified the principal diffusion direction, whereas BPX returned approximately uniform density of diffusion directions at all angles (i.e., little to no angular information). BaMM consistently estimated two diffusion directions with > 100 DWI. BPX consistently estimated two directions with > 20 DWI. Angular measurement error was generally less with BPX than BaMM, but comparable for > 250 DWI. When BPX max fiber count was increased to 3 (Figure S20), BPX estimated three fibers with > 250 DWI, and angular measurement error increased for all sample sizes.

CSA-QBI most frequently estimated three fiber directions at all sub-sampling sizes, but also frequently estimated one or two fibers. The CSA-QBI estimation of two or three fibers was broadly distributed over 0 to *π* for < 500 DWI, and the error improved only marginally with increasing DWIs.

CSD also frequently estimated three fiber directions with < 30 DWI and with > 300, <800 DWI. When CSD estimated two fiber directions, the angle estimations matched that of BaMM and BPX.

### 3.7. Right corticospinal tract: poor reliability and non-converging fiber count

The third ROI we analyzed in depth was in the right corticospinal tract (CST) as it progressed through/near the internal capsule, a brain region with potentially three crossing fibers (MNI 22, −19, 11; Figure S25A). Based on anatomical priors, model sensitivity, registration to MNI coordinates, and accuracy of ROI location across subjects, we could expect a single fiber direction reflecting the CST, two fiber directions for the CST and internal capsule, or three directions for a fanning behavior of either the CST or internal capsule fibers. BPX settings were set to a maximum of three fibers accordingly. Results for Subjects 1 and 3 are in Figures S26-27, respectively. Reliability curves for all methods and subjects (including LLS and STB) are shown in Figure S28

BaMM, CSA-QBI, and CSD estimated varying number of fibers with different sample sizes, while BPX estimated three fibers with almost uniform angular distribution of *ϕ* and *θ* from 0 to *π*. Estimated error (relative to the mean angle orientation estimated by that method using all available data) improved with increasing number of DWI for all methods. However, since the models diverged in their estimation of number of fibers and the orientation of those fibers, we can only speak to the reliability of the models relative to themselves and not their accuracy.

### 3.8. Repeated ABCD scans as reliable as single session high angular resolution scan

In response to the concern that repeated ABCD scans increased angular sampling in random rather than controlled fashion, we collected single session high angular resolution (SS-HAR) data. The sequence replicated the ABCD scan used previously and replaced the 96-direction vector set with a 960-direction vector set. Subject 1 was rescanned using this sequence that had a total of 1020 DWI, replicating the ratio of diffusion weighted volumes to b0 images in the original sequence. All methods generally replicated trends observed previously (Figure S29), with equivalent reliability when comparing the single scan and repeated sampling approaches.

## 4. Discussion

Identifying and understanding inter-individual differences in brain organization is critically important for neuroscience, neurology, neu-rosurgery, and psychiatry (Fair et al., 2021; Gordon et al., 2017; Gratton et al., 2020; Mitchell et al., 2013). While almost all typically developing individuals share the same major white matter bundles (Mori et al., 2005), variations in size, position, and/or orientation of white matter fibers could have significant effects on surgical plans (Luque Laguna et al., 2020; Roland et al., 2021), and recovery from brain injury (Laumann et al., 2021).

### 4.1. Reliability and limited accuracy of classic single tensor fitting (LLS, STB)

In both highly sampled human and simulated data, single tensor estimate variability (LLS, STB) decreased with increasing sample size. Assuming a normal distribution, measurement error should be inversely related to the square root of the sample size (e.g., DWI directions in this context). Our results failed to follow this pattern under two conditions: when there were insufficient data to constrain the model (e.g., < 20 DWI directions for LLS and STB), or when the model misrepresented the underlying diffusion process (i.e., using single tensor methods for multiple fibers, or assuming excess fibers for a single fiber direction). Overall, deep white matter voxels showed lower measurement error than the rest of the brain, and larger data amounts were needed for voxels with lower FA (Fig. 6). FA measurement error was < 5% with 70–150 DWIs in deep white matter, while cortical voxels required 300–500 DWIs to comparably reduce error. Angles *ϕ* and *θ*, which are critical for tracking applications, showed the highest measurement error of all the diffusion metrics. Uncertainty in the angle of the tensor is related to uncertainty in anisotropy, explaining why angle error is higher in gray matter (Jones and Cercignani, 2010).

The accuracy of single-tensor modeling in regions of crossing fibers is inherently limited because the model cannot accurately represent the underlying diffusion process. Improved accuracy can be achieved with more complex models. Yet, more complex models increase the likeli-hood of over fitting and thus require additional testing and validation to ensure that biases inaccurate assumptions are avoided.

### 4.2. BaMM: a novel estimation method for preventing overfitting of diffusion data

Bayesian methods provide a useful approach for this type of problem by incorporating model selection in the analysis, which minimizes the risk of over fitting by incorporating a penalty against models that use an excessive number of parameters. The Bayesian Multi-tensor Model-selection (BaMM) method was designed in accordance with this principle. The BaMM parameter estimation algorithm is based on previous Bayesian, model-selection methods (Bretthorst, 1990). We designed BaMM to compare multiple diffusion models and select the model best suited for the available data. Here, we demonstrate that BaMM can accurately estimate zero, one, two, or three sticks, and that its precision improves with increasing DWI data. In the current implementation, BaMM uses the same assumed model as BPX (ball-and-sticks; (Behrens et al., 2003)) but can accommodate a large input data set by scaling the parameter estimation penalties to the dataset size. We tested and validated BaMM over a wide range of diffusion measurements (10 to 1000) to rule out bias for a specific number of DWIs. The current work was completed using a ball- and- sticks partial volumes model, but BaMM can also accommodate full multi-tensor models, multi-fiber kurtosis models, or other models yet to be developed (Chiang et al., 2019; Wang et al., 2014). The BaMM framework is adaptable to any set of mathematical assumptions about white matter structure and can serve to directly compare different diffusion models against the available data.

### 4.3. BPX: accurate and reliable only if assumptions are met

BPX was in the past one of the most popular schemes for probabilistic tracking. BPX was initially published with 30 direction DWI data, and then with 60 direction DWI data (Jbabdi et al., 2012; Sotiropoulos et al., 2013). The datasets analyzed here contained much higher quantities of data (800+ direction low-motion DWIs for each individual). With an excess of DWIs, we observed that BPX consistently estimates the maximum allowed number of fibers. BPX’s default setting is a maximum of two fibers, and with these settings BPX estimated two fibers for simulated single tensor data (Fig. 3) and for the corpus callosum (Fig. 7). When set to allow for a maximum of three tensors, BPX estimated three fibers in simulated single tensor data and in the corpus callosum ROI (Figure S4 and S17). When provided with large data sets (> 500 DWI), BPX tends to split a single tensor into two that are almost superimposed. This is inaccurate but likely not detrimental to subsequent tractography. By contrast, a potentially inappropriately oriented second or third fiber, could substantially deviate probabilistic tracking (Fig 9C-D). When BPX’s assumptions are met, it is accurate and reliable from 10 to 1000 DWIs, but determining the appropriate priors for all brain voxels poses a significant challenge.

### 4.4. CSA-QBI: accurate only with very large amounts of diffusion data

Constant solid angle Q-Ball Imaging (CSA-QBI) was designed to eliminate diffusion tensor shape assumptions. CSA-QBI is a method derived from q-space formalism (Callaghan et al., 1988) and uses a Funk-Radon transform to estimate the ODF (Aganj et al., 2010; Tuch, 2004). CSA-QBI can estimate one, two or three tensors with 1000 DWIs, but problematically, the reliability of these estimates always remained low (Fig 3-5, 7-9). With < 800 DWIs QBI tends to model additional fiber directions, possibly capturing noise in the data. CSA-QBI requires many more DWI than what is currently being acquired in a clinical or research setting.

### 4.5. CSD improved fiber ODF estimation with sufficient data

Constrained Spherical Deconvolution (CSD) from the imaging package MRtrix3 (Tournier et al., 2019) is another method that uses spherical harmonic deconvolution. In contrast to CSA-QBI, CSD uses a constrained spherical deconvolution to estimate the fiber ODF. CSD demonstrated greater reliability with smaller sampling density than CSA-QBI. Yet a subset of permutations often estimated excess fiber directions. The ABCD acquisition scheme (Casey et al., 2018) used in the current analyses achieves high angular resolution diffusion imaging, yet CSD often necessitated larger quantities of DWI for consistent fiber direction estimation. In aggregate, the results of the present CSA-QBI and CSD testing suggest that precise and reliable fiber estimation may require greater quantities of data than are typically obtained.

### 4.6. Overfitting and prior dependence of multi-fiber diffusion imaging methods

The last twenty years of diffusion imaging research have generated a steady progression of new and increasingly complex models (Aganj et al., 2010; Behrens et al., 2003; Jbabdi et al., 2012; Tournier et al., 2007, 2013; Tuch, 2004; Wang et al., 2014). The most novel and potentially exciting methodologies may have outstripped the conventionally acquired quantity of data needed to constrain the model. In the current work, we tested a broad distribution of the most widely cited methodologies and parameter estimation approaches: the classic diffusion tensor model that endures due to its simplicity (Basser et al., 1994a, Basser et al., 1994b; Pierpaoli et al., 1996); FSL’s BedpostX that popularized the ball and stick model, which simplifies shape assumptions (Behrens et al., 2007, 2003; Jbabdi et al., 2012; Jenkinson et al., 2012); DSI Studio’s Constant Solid-Angle Q-Ball Imaging which was derived from q-space formalism (Callaghan et al., 1988) and estimates the ODF (Aganj et al., 2010; Tuch, 2004); and finally MRtrix3’s Constrained Spherical Deconvolution that estimates the fiber ODF (Tournier et al., 2007, 2013, 2019). While this list is not exhaustive and novel methods will continue to be developed, the models tested here similarly share a sensitivity to inappropriate priors and vulnerability to overfitting. Underrecognition of this point may underlie an emphasis on complex models and a relative underemphasis on the quantity and quality of DWI data needed to achieve accurate fiber estimation.

### 4.7. High angular resolution diffusion data acquisition

In comparison to the repeated sample acquisitions, we also collected supplementary DWI data with 960 unique B-vectors and 50 b0 applied (1020 DWI). Of the methods tested in the current work, none of them improved with single-scan high angular resolution data, although CSD performed equally well with both data acquisition schemes (Figure S29). Instead, it appears that repeated acquisitions of ABCD’s 103 DWI protocol were potentially less prone to overfitting. Combining DWI samples over multiple sessions introduces jitter owing to variability of head position and effectively improves angular sampling.

### 4.7. Precision diffusion imaging is achievable with practical data acquisition times

Only 15 to 30 DWIs are typically acquired in clinical settings. As MRI hardware and processing software improved, researchers started to acquire larger diffusion data sets (100 - 300 DWIs per subject) while maintaining reasonable imaging times (<20 min). Our study demonstrated that one can reliably estimate the shape and orientation of a single diffusion tensor in deep white matter with about 100 diffusion measurements. Thus, researchers (Casey et al., 2018; Paquette et al., 2016; Van Essen et al., 2012) as well as clinicians ones should consider collecting a greater number of DWIs (at least ~100) than has been typical.

For crossing-fiber diffusion models, at least 300 DWIs are generally required in deep white matter, assuming high data quality. To advance from 100 to 300 DWI requires an increase in total scan time from about 6 min to about 20 min. Acquiring 1000 DWIs with current technology takes a little over an hour. An hour- long diffusion scan may be warranted for precision mapping for research or in neurosurgical planning (Conti Nibali et al., 2019). Diffusion data acquisition is typically better tolerated than task or resting state functional MRI (fMRI) because the patient can sleep or watch a movie during the scan. Therefore, additional investment in scanning time could have significant positive effects on diagnostics and treatment of neurological and neurosurgical patients. In addition, acquiring greater amounts of high-quality DWI data would expand the available processing schemes beyond the models described here to methodologies which require even more data (e.g., DSI, DBSI) (Paquette et al., 2016; Wang et al., 2015).

### 4.8. Structural connectivity maps (end-to-end tracking) in cortex

Researchers have been exploring the feasibility and validity of MRI-based structural connectivity analyses for decades (Baum et al., 2018; Maier-Hein et al., 2017; Messaritaki et al., 2019; Pestilli et al., 2014; Roine et al., 2019; Satterthwaite et al., 2013; Sotiropoulos and Zalesky, 2019; Yeh et al., 2018). Many studies that attempt to build structural connectivity maps initiate the fiber tracking at the border of gray and white matter. Since FA and angle orientations (*ϕ* and *θ*; Fig. 6, S13) are less reliable closer to gray matter, more errors are introduced at initiation of the tracking. Although many other challenges to structural connectivity maps must still be addressed (Jespersen et al., 2007; Van Essen et al., 2014), structural connectivity and other advanced modeling techniques would likely also greatly benefit from larger numbers of DWIs per individual.

### 4.9. Summary: accuracy and reliability of diffusion imaging models

We evaluated the accuracy and reliability of single and multiple crossing-fiber models (LLS, STB, BPX, CSA-QBI, CSD, BaMM) in both simulated and repeatedly sampled human DWI data (9 - 14 complete DWI datasets), as a function of data amount. LLS, STB and BPX were only capable of reifying their prior assumptions independent of data amount. CSA-QBI required very large numbers of DWIs (>800) to start approaching a degree of reliability and accuracy. CSD and BaMM performed much better, with BaMM proving the relatively most over fitting resistant across test cases. To enhance the scientific and clinical utility of diffusion imaging, more data should be collected per individual and analyses should be conducted with methods designed to reduce overfitting.

## Supplementary Material

Supplementary Material

## Figures and Tables

**Table 1:** Summary of methods and reliability comparisons.

Method	Linear Least Squares (LLS)	Single Tensor Bayesian (STB)	FSL’s BedpostX (BPX)	Bayesian Multi-Tensor Model-Selection (BaMM)	DSI’ Constant Solid Angle Q-Ball Imaging (CSA-QBI)	MRtrix3’s Constrained Spherical Deconvolution (CSD)
Model	Single tensor	Single tensor with Biological Priors	Sum isotropic and *N* fiber compartments, automatic relevance detection	Sum isotropic and *N* fiber compartments, Bayesian model selection	Diffusion orientation distribution function (dODF) with constant solid angle	Fiber orientation distribution (FOD) through constrained spherical deconvolution
Simulated Single Tensor	SNR 30, 50, 100	SNR 30, 50, 100	SNR 30, 50, 100	SNR 30, 50, 100	SNR 30, 50, 100	SNR 30, 50, 100
Simulated Two Crossing Tensors	–	–	SNR 30, 50, 100 Fraction 50/50, 60/40, 80/20% FA 0.6, 0.8 Angle 30°, 60°, 90°	SNR 30, 50, 100 Fraction 50/50, 60/40, 80/20% FA 0.6, 0.8 Angle 30°, 60°, 90°	SNR 30, 50, 100 Fraction 50/50, 60/40, 80/20% FA 0.6, 0.8 Angle 30°, 60°, 90°	SNR 30, 50, 100 Fraction 50/50, 60/40, 80/20% FA 0.6, 0.8 Angle 30°, 60°, 90°
Simulated Three Crossing Tensors	–	–	SNR 30, 50, 100 Fraction 33/33/33, 40/34/26, 53/34/13% FA 0.6, 0.7, 0.8 Angle 30°, 60°, 90°	SNR 30, 50, 100 Fraction 33/33/33, 40/34/26, 53/34/13% FA 0.6, 0.7, 0.8 Angle 30°, 60°, 90°	SNR 30, 50, 100 Fraction 33/33/33, 40/34/26, 53/34/13% FA 0.6, 0.7, 0.8 Angle 30°, 60°, 90°	SNR 30, 50, 100 Fraction 33/33/33, 40/34/26, 53/34/13% FA 0.6, 0.7, 0.8 Angle 30°, 60°, 90°
Human Brain Reliability Map	All three subjects	–	–	–	–	–
Reliability Curve on Specific ROI	All three subjects	All three subjects	All three subjects	All three subjects	All three subjects	All three subjects

## Data Availability

The neuroimaging data generated during this study will be made available on OpenNeuro before publication. All code needed to reproduce our analyses will be made available on Gitlab before publication.
