# Supplementary material for: Accuracy and reliability of diffusion imaging models
Source: Neuroimage. Author manuscript; Available in PMC 2023 Jan 16. (PMC9841915; doi:10.1016/j.neuroimage.2022.119138)
Supplement: Supplementary Material [file NIHMS1840704-supplement-Supplementary_Material.docx]

**Supplemental Methods:**

Bayesian Multi-Tensor Model-Selection (BaMM): The Bayesian model selection algorithm was followed by parameter estimation of the winning model (modified from (Lee et al., 2010)). Specifically, posterior probabilities for all model parameters were computed voxel by voxel, using diffusion weighted imaging data with Bayesian probability theory (Jaynes, 2003). Using the ball-and-sticks model (Eq.1), there were eleven parameters per voxel, expressed in groups $\Omega_{i}$ with voxel indices $i$:

$\Omega_{i}=\left\{ S_{0},d,f_{1},f_{2},f_{3},\phi_{1},\phi_{2},\phi_{3},\theta_{1},\theta_{2},\theta_{3} \right\}$ [Eq. S1]

Two parameters describe the ball signal with $B_{0}$ and the ball isotropic diffusion ($S_{0},d$). Three parameters describe the respective stick fractions $(f_{1},f_{2},f_{3})$, and six parameters describe the orientation of the respective sticks $\left( \phi_{1},\phi_{2},\phi_{3},\theta_{1},\theta_{2},\theta_{3} \right)$.

Bayes’ theorem states that the posterior probability for the parameters, given data, $D_{i}$, and relevant background information, $I$, can be expressed as (Jaynes, 2003):

$P\left( \Omega_{i} | D_{i},I \right)\propto P\left( D_{i} | \Omega_{i},I \right)P\left( \Omega_{i} | I \right)$*,*  [Eq. S2]

For which $P\left( D_{i} | \Omega_{i},I \right)$ is the likelihood of the data given the model parameters and $P\left( \Omega_{i} | I \right)$ is the prior probability for the model parameters. The prior probability was factored into independent prior probabilities for each parameter:

$P\left( \Omega_{i} | I \right)\propto P\left( S_{0} | I \right)P\left( d | I \right)P\left( f_{1} | I \right)P\left( f_{2} | I \right)P\left( f_{3} | I \right)P\left( \phi_{1} | I \right)P\left( \phi_{2} | I \right)P\left( \phi_{3} | I \right)P\left( \theta_{1} | I \right)\ldots$ [Eq. S3]

All prior probabilities were assigned Gaussian distributions bounded by appropriate physiologic ranges. The ranges selected for the parameters in our data set were $S_{0}$ (0-1.5*max[input data]); $d$(0-6 x 10^-3^ mm^2^/sec); $f_{1-3}$ (0-1.0, dimensionless); $\phi_{1-3}$ and $\theta_{1-3}$ (0-$\pi$ radians). The likelihood for the data from a voxel, given the model parameters $P\left( D_{i} | \Omega_{i},I \right)$ is expressible as a marginal probability when the standard deviations, $\sigma_{i}$, are removed using the sum and product rules:

$P\left( D_{i}\left| \Omega_{i} \right|I \right)= \int P\left( \sigma_{i},D_{i} | \Omega_{i},I \right)d\sigma_{i}\propto\int P(\sigma_{i}\left| I \right)P\left( D_{i} | \sigma_{i},\Omega_{i},I \right)d\sigma_{i}$ [Eq. S4]

Assigning Jeffreys’ prior probability to $P\left( \sigma_{i} | I \right)$ and assigning the Gaussian distribution to $P\left( D_{i} | \sigma_{i},\Omega_{i},I \right)$, the marginal probability for the data may be written as the Student’s *t* distribution:

$P\left( D_{i} | \Omega_{i},I \right)\propto\frac{1}{2}\Gamma\left( \frac{M}{2} \right)\left( \frac{Q_{i}}{2} \right)^{-M/2}$ [Eq. S5]

$M$ is the number of diffusion samples per voxel. $Q_{i}$ is the total squared residual:

$Q_{i}=\sum_{k=1}^{M} \left( E_{ik}-S_{ik} \right)^{2}$ [Eq. S6]

$E_{ik}$ is the measured signal and $S_{ik}$ is the model estimated signal. The join posterior probability was estimated using Markov-chain Monte Carlo, Metropolis-Hastings sampling, and simulated annealing.

**References:**

Lee, J.J., Bretthorst, G.L., Derdeyn, C.P., Powers, W.J., Videen, T.O., Snyder, A.Z., Markham, J., and Shimony, J.S. (2010). Dynamic susceptibility contrast MRI with localized arterial input functions. Magnetic resonance in medicine *63*, 1305-1314.

Jaynes, E.T. (2003). Probability Theory: The Logic of Science (Cambridge: Cambridge University Press).

**Supplemental Figures:**

**
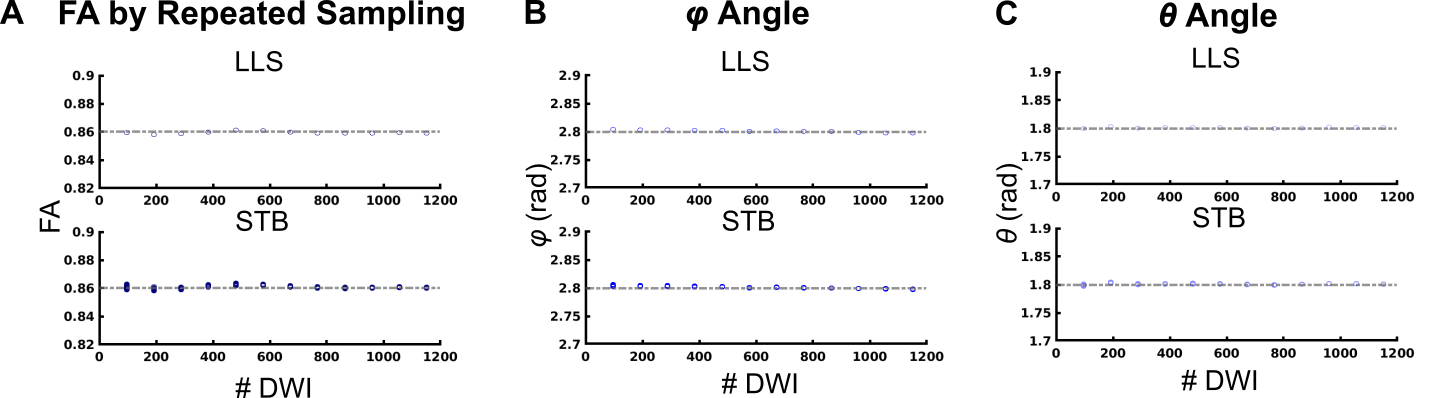
**

**Supplementary Figure 1:** **Validation of Subsampling in Subject 2**

Repeated sampling by selecting an entire acquisition of 103 DWI in Subject 2 (aka, randomly select 1-12 acquisitions). (A) FA by Linear Least Squares (LLS) and Single Tensor Bayesian (STB) with repeated sampling of 1-12 acquisitions. (B) Angle $\phi.$ (C) Angle $\theta$


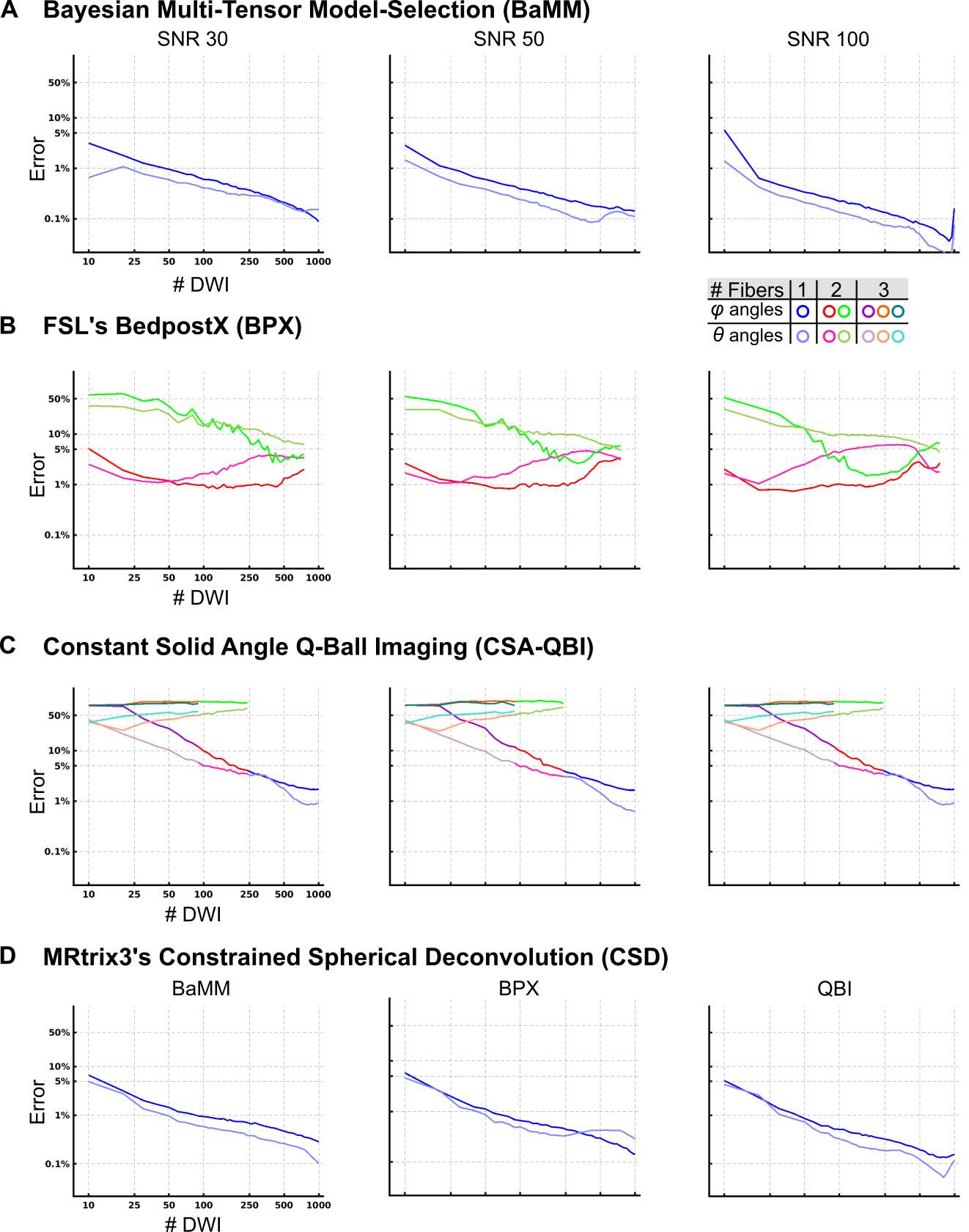


**Supplementary Figure 2: Accuracy of Diffusion Measures, Simulated Single Tensor**

(A) Error estimation by Bayesian Multi-tensor Model-selection (BaMM) at SNR30, SNR50, and SNR100, Mean error at each subsampling size was calculated, then plotted on a log scale. Plots are colored by the most frequent number of fibers estimated: subsamples with a single fiber direction are plotted in blue/sky blue, two fibers plotted red/pink and green/olive, 3 fibers are plotted in purple/lilac, orange/salmon, teal/cyan. (B) BedpostX (BPX), (C) Constant Solid Angle Q-Ball Imaging (QBI), (D) Constrained Spherical Deconvolution.


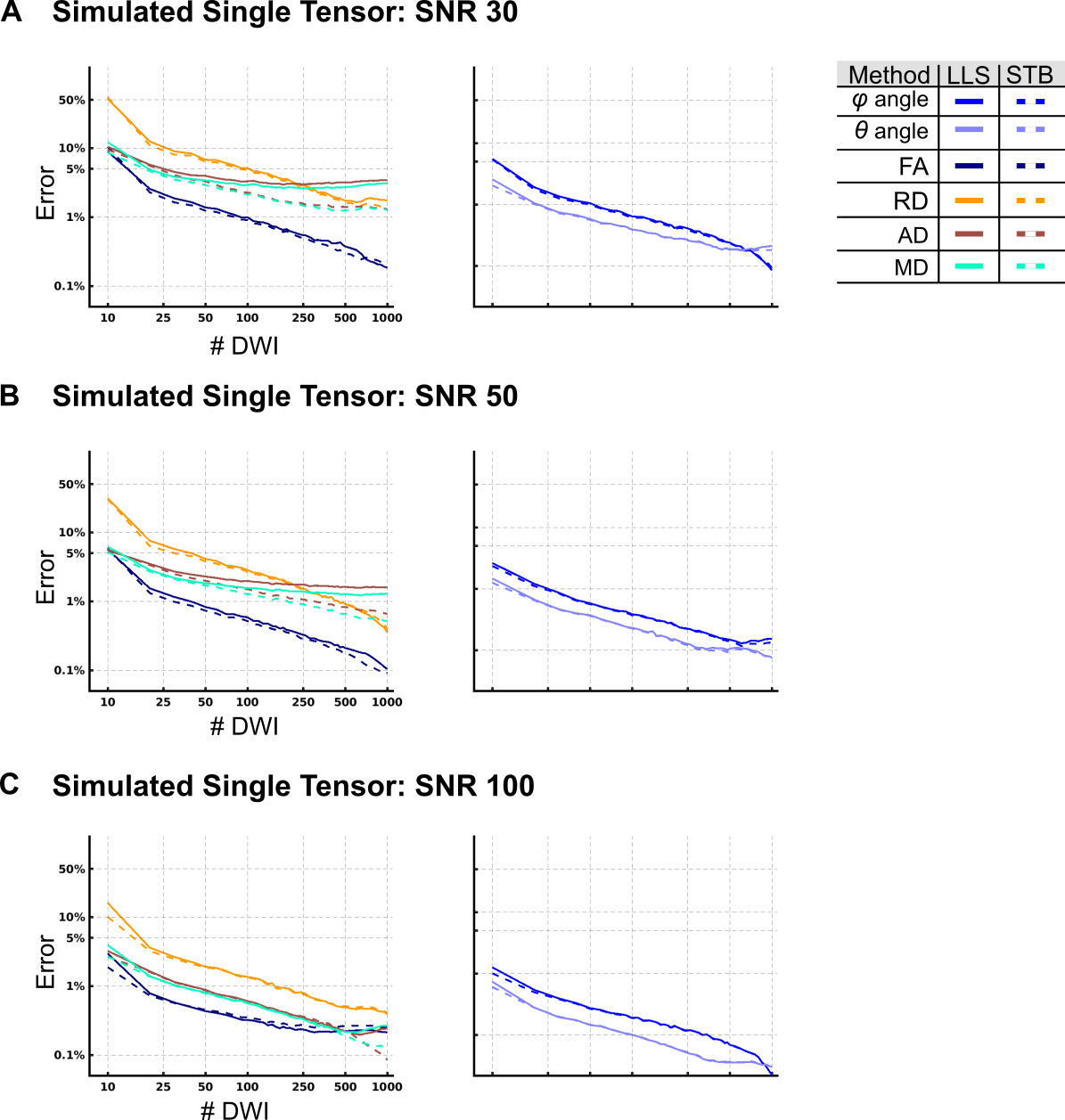


**Supplementary Figure 3: Accuracy of DTI Measures by LLS and STB, Simulated Single Tensor**

(A) (A) Error estimation by Linear Least Squares (LLS) and Single Tensor Bayesian (STB) at SNR30. Mean error at each subsampling size was calculated, then plotted on a log scale. LLS and STB plotted using straight and dotted lines, respectively. FA is in navy, RD in yellow, AD in brown, and MD in bright green. Phi is plotted in blue, and theta in sky blue. (B) SNR 50. (C) SNR 100.


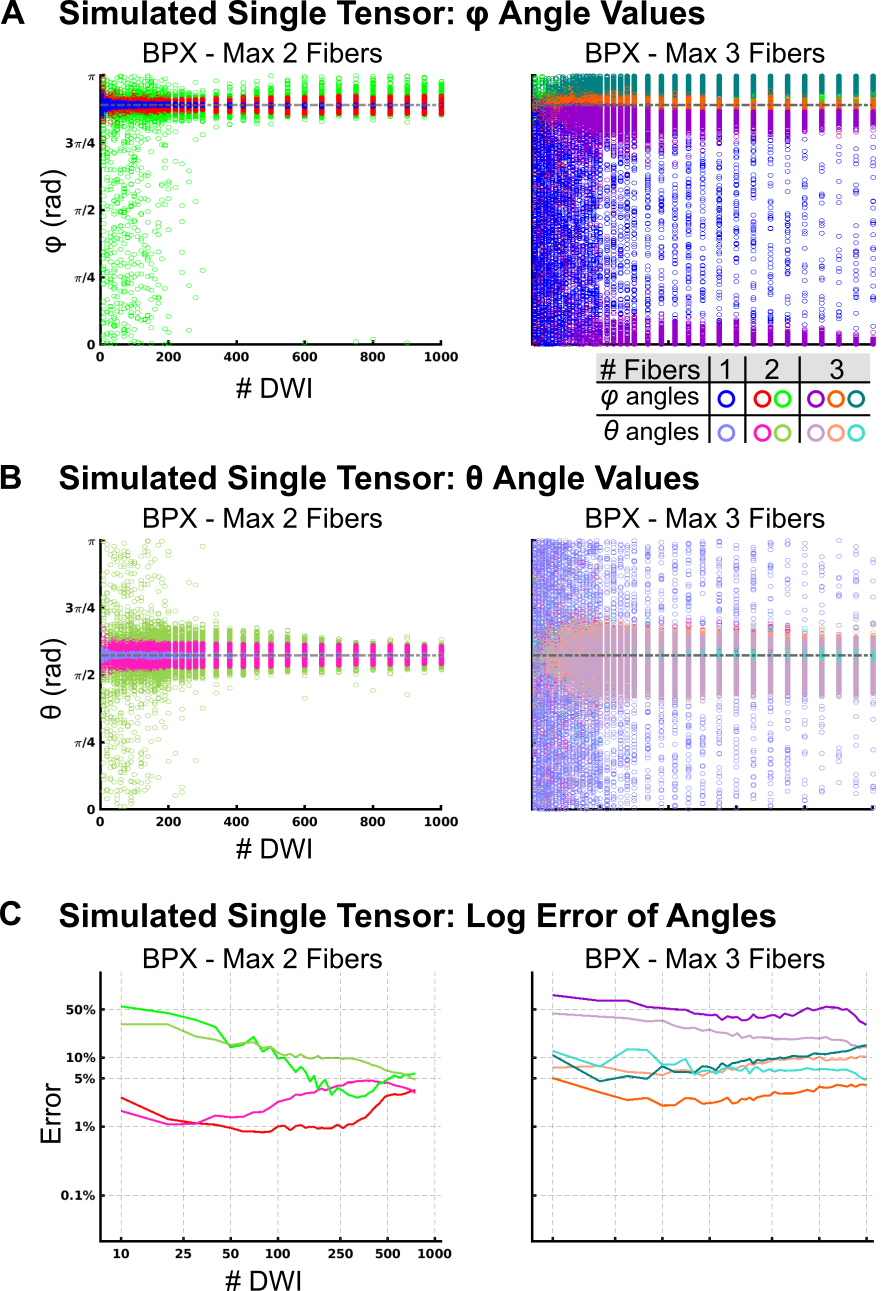


**Supplementary Figure 4: Accuracy of Diffusion Measures by BPX, Simulated Single Tensor with Max 3 Fibers**

(A) φ angle estimations by BedpostX (BPX) with maximum 2 or 3 fibers. Open circles represent the results obtained by repeated permutation sampling. Same color legend for all data panels. Permutations that resulted in a single fiber direction are plotted in blue (φ). Permutations that resulted in two fibers are plotted in red (φ) and green (φ). Permutations that resulted in three fibers are plotted in purple (φ), orange (φ), teal (φ). (B) θ angle estimations. Permutations are plotted in sky blue (θ) for one fiber, pink (θ) and olive (θ) for two fibers, and lilac (θ), salmon (θ), cyan (θ) for three fibers. (C) Error estimation for BPX with max 2 or 3 fibers. Mean error at each subsampling size was calculated, then plotted on a log scale. The same colors as in (A/B) are used and indicate the most frequent number of fibers estimated at each subsampling size.


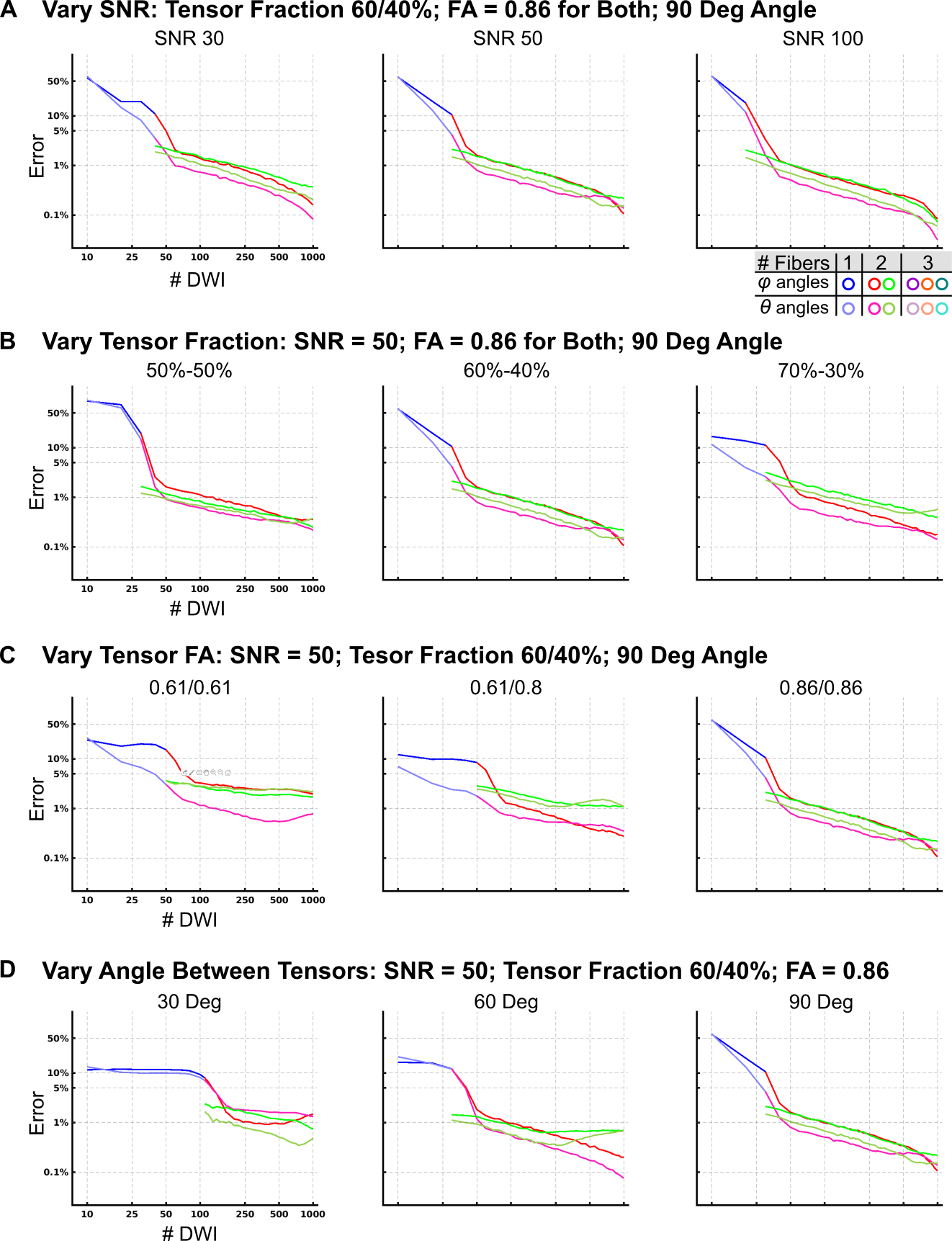


**Supplementary Figure 5: Accuracy of BaMM in variety of two tensor simulated data**

(A) Error estimation by BaMM for two crossing tensors at varying SNR. Mean error at each subsampling size was calculated, then plotted as the log of error. Plots are colored by the most frequent number of fibers estimated: subsamples with a single fiber direction are plotted in blue/sky blue, two fibers plotted red/pink and green/olive, 3 fibers are plotted in purple/lilac, orange/salmon, teal/cyan. (B) Vary tensor Fraction. (C) Vary tensor FA. (D) Vary angle between tensors.


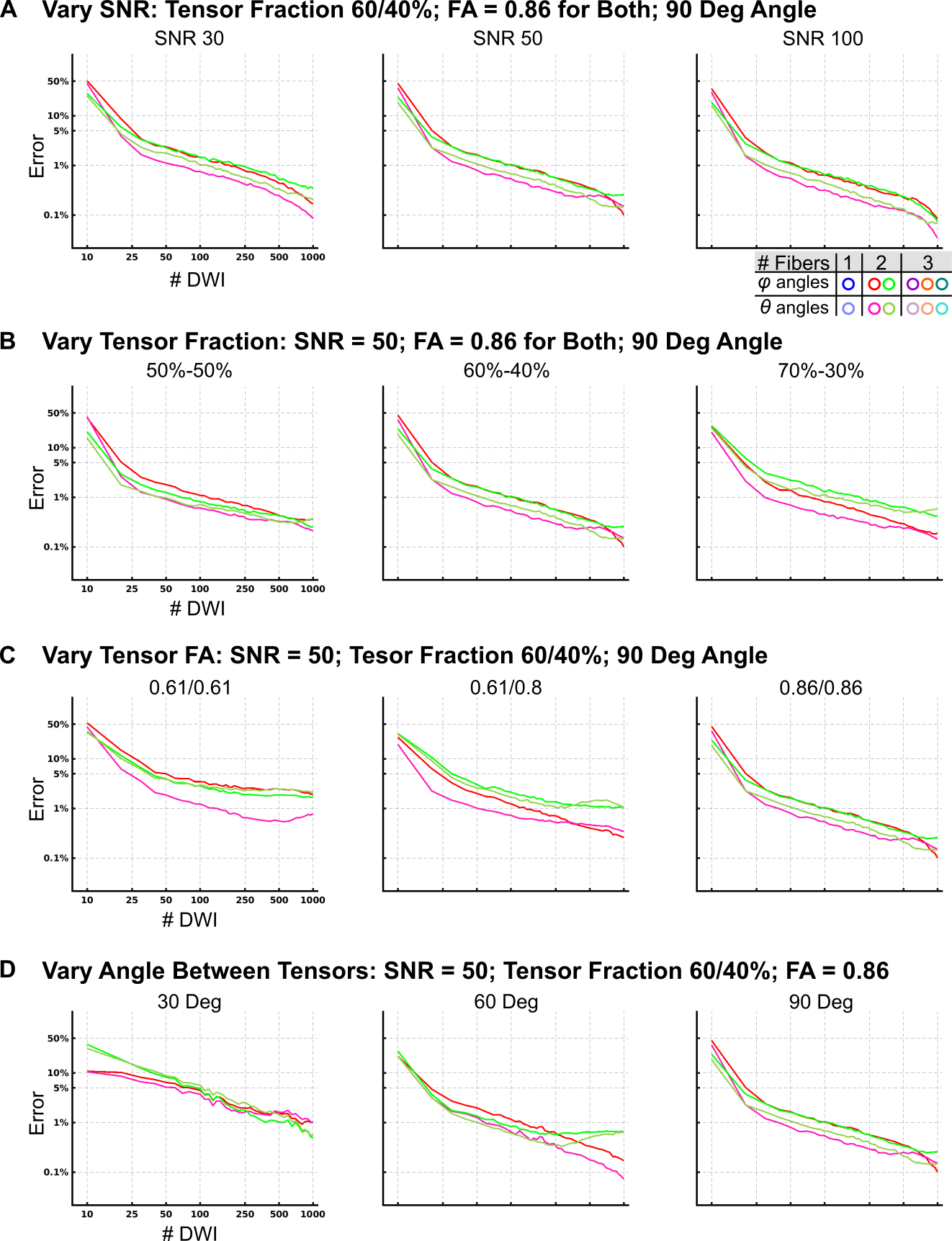


**Supplementary Figure 6: Accuracy of BPX in variety of two tensor simulated data**

(A) Error estimation by BPX for two crossing tensors at varying SNR. Mean error at each subsampling size was calculated, then plotted as the log of error. Plots are colored by the most frequent number of fibers estimated: subsamples with a single fiber direction are plotted in blue/sky blue, two fibers plotted red/pink and green/olive, 3 fibers are plotted in purple/lilac, orange/salmon, teal/cyan. (B) Vary tensor Fraction. (C) Vary tensor FA. (D) Vary angle between tensors.

**
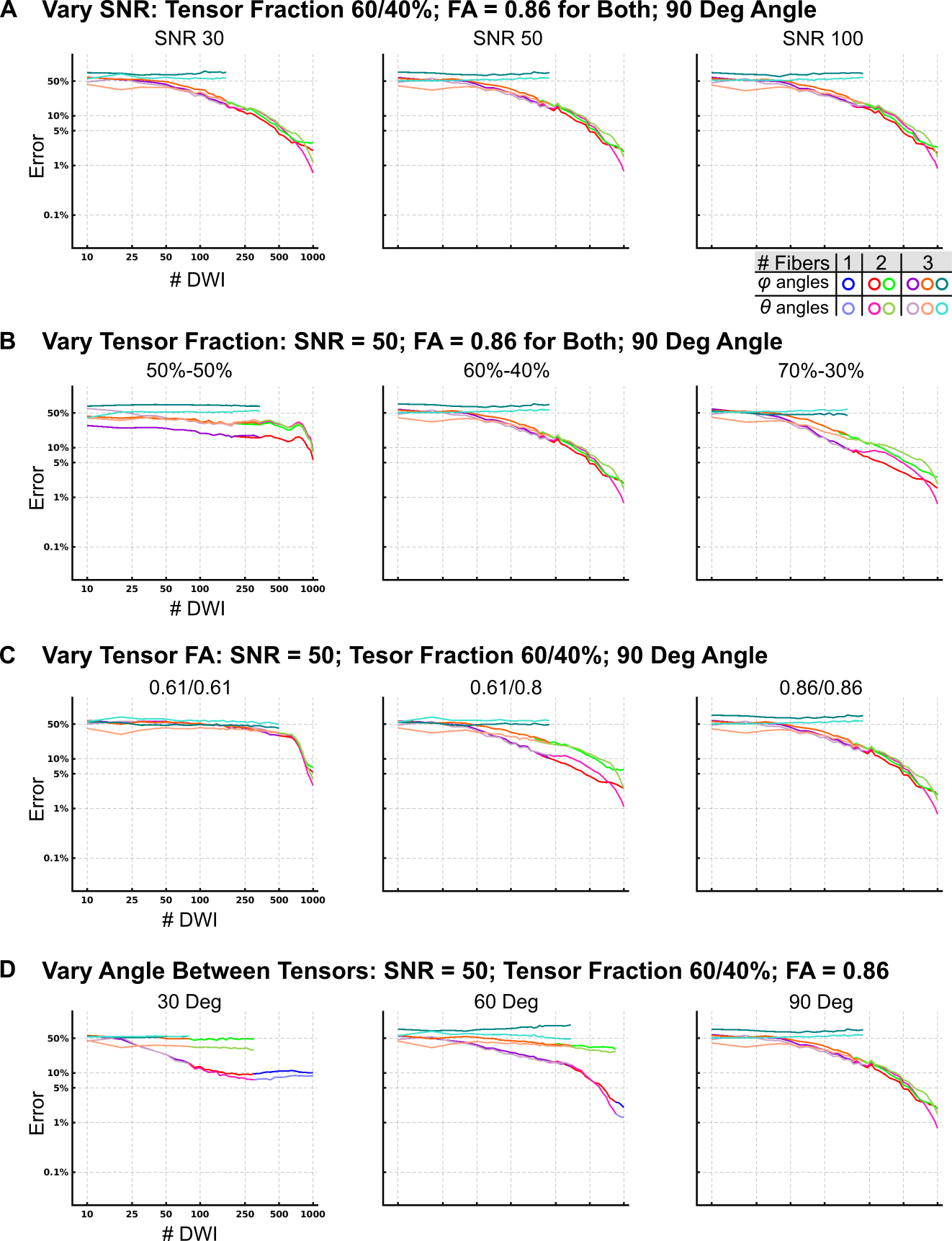
**

**Supplementary Figure 7: Accuracy of CSA-QBI in variety of two tensor simulated data**

(A) Error estimation by CSA-QBI for two crossing tensors at varying SNR. Mean error at each subsampling size was calculated, then plotted as the log of error. Plots are colored by the most frequent number of fibers estimated: subsamples with a single fiber direction are plotted in blue/sky blue, two fibers plotted red/pink and green/olive, 3 fibers are plotted in purple/lilac, orange/salmon, teal/cyan. (B) Vary tensor Fraction. (C) Vary tensor FA. (D) Vary angle between tensors.

**
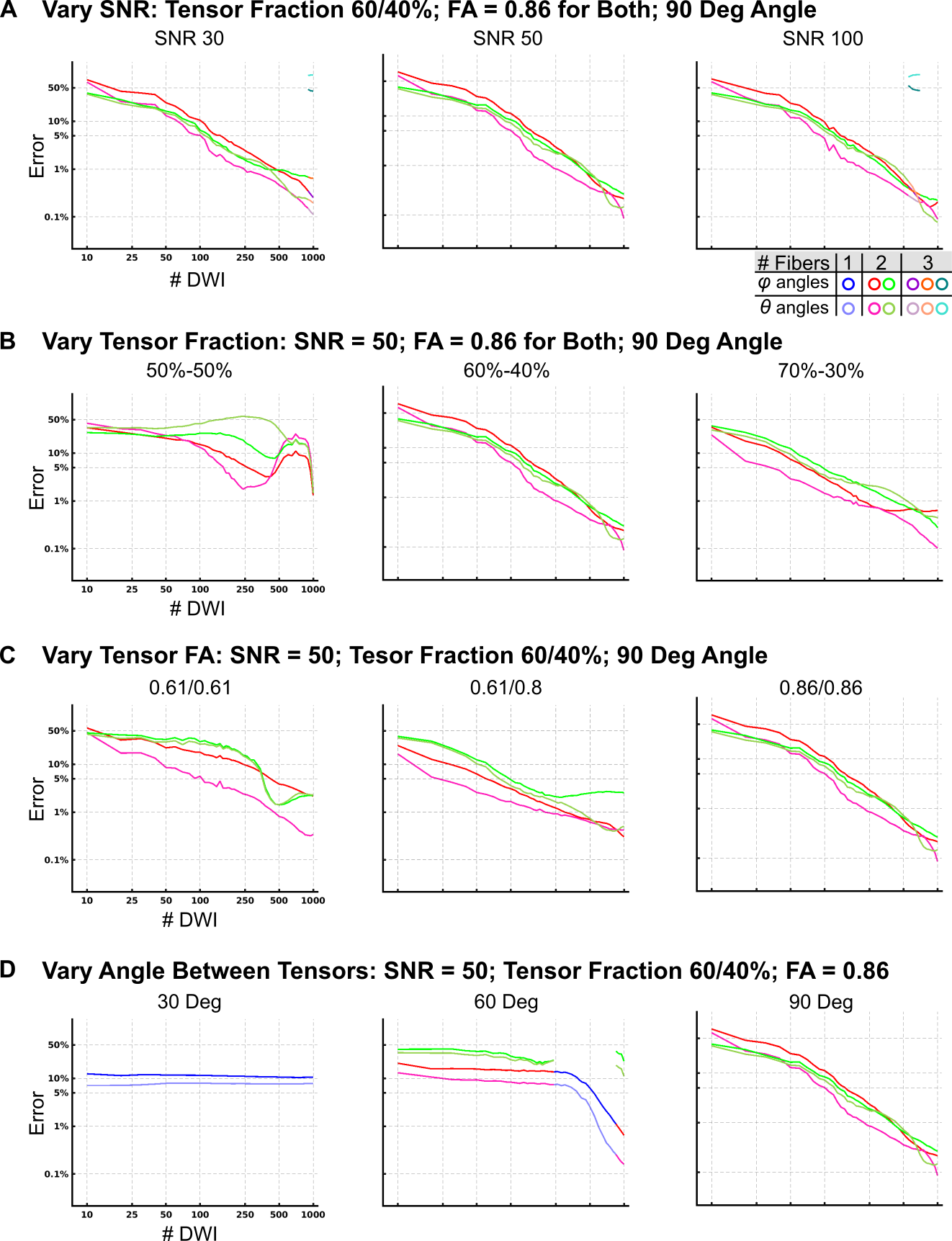
**

**Supplementary Figure 8: Accuracy of CSD in variety of two tensor simulated data**

(A) Error estimation by CSD for two crossing tensors at varying SNR. Mean error at each subsampling size was calculated, then plotted as the log of error. Plots are colored by the most frequent number of fibers estimated: subsamples with a single fiber direction are plotted in blue/sky blue, two fibers plotted red/pink and green/olive, 3 fibers are plotted in purple/lilac, orange/salmon, teal/cyan. (B) Vary tensor Fraction. (C) Vary tensor FA. (D) Vary angle between tensors.


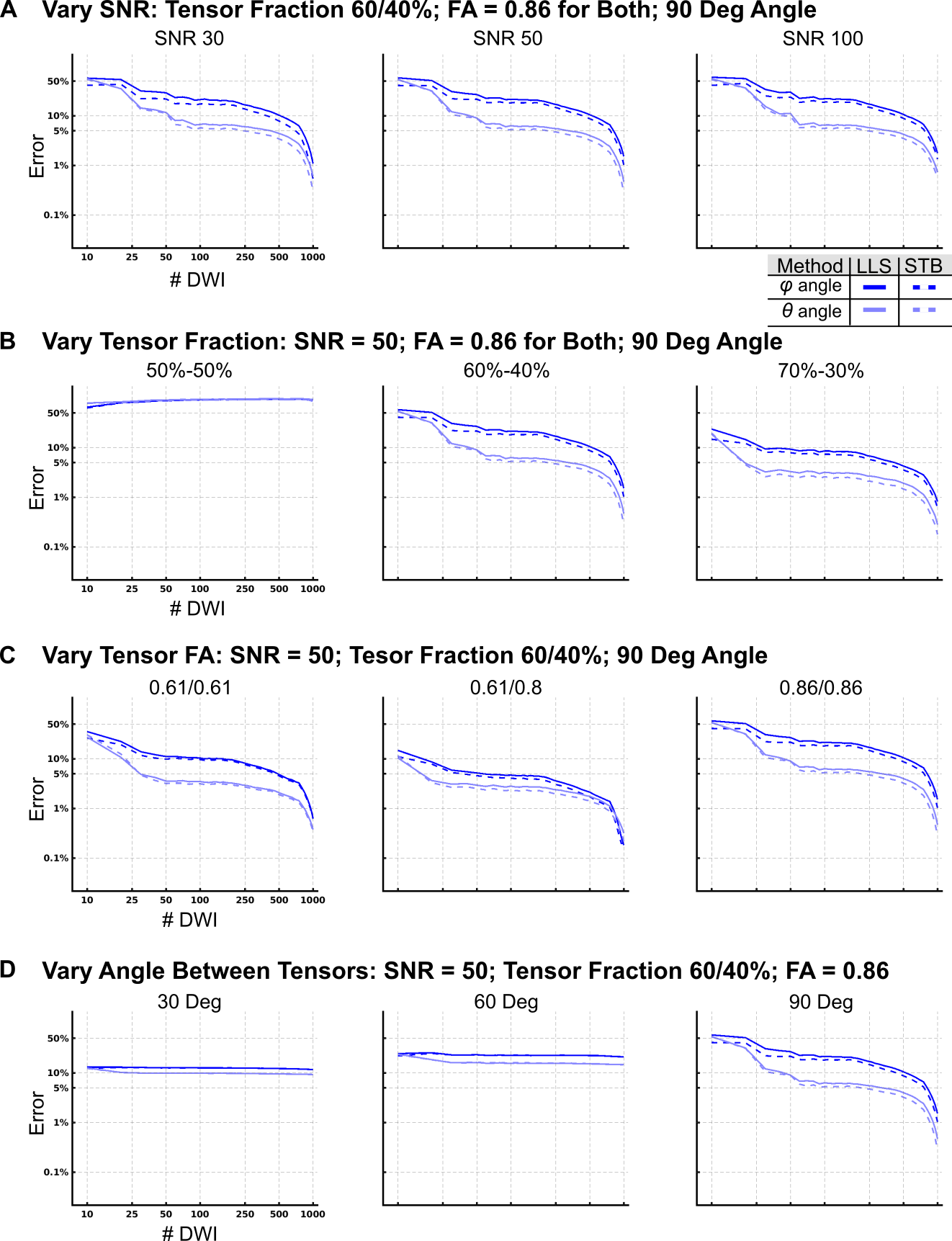


**Supplementary Figure 9: Accuracy of LLS and STB in variety of two tensor simulated data**

(A) Error estimation by LLS and STB for two crossing tensors at varying SNR. Mean error at each subsampling size was calculated, then plotted as the log of error. Plots are colored by the most frequent number of fibers estimated: subsamples with a single fiber direction are plotted in blue/sky blue, two fibers plotted red/pink and green/olive, 3 fibers are plotted in purple/lilac, orange/salmon, teal/cyan. (B) Vary tensor Fraction. (C) Vary tensor FA. (D) Vary angle between tensors.

**
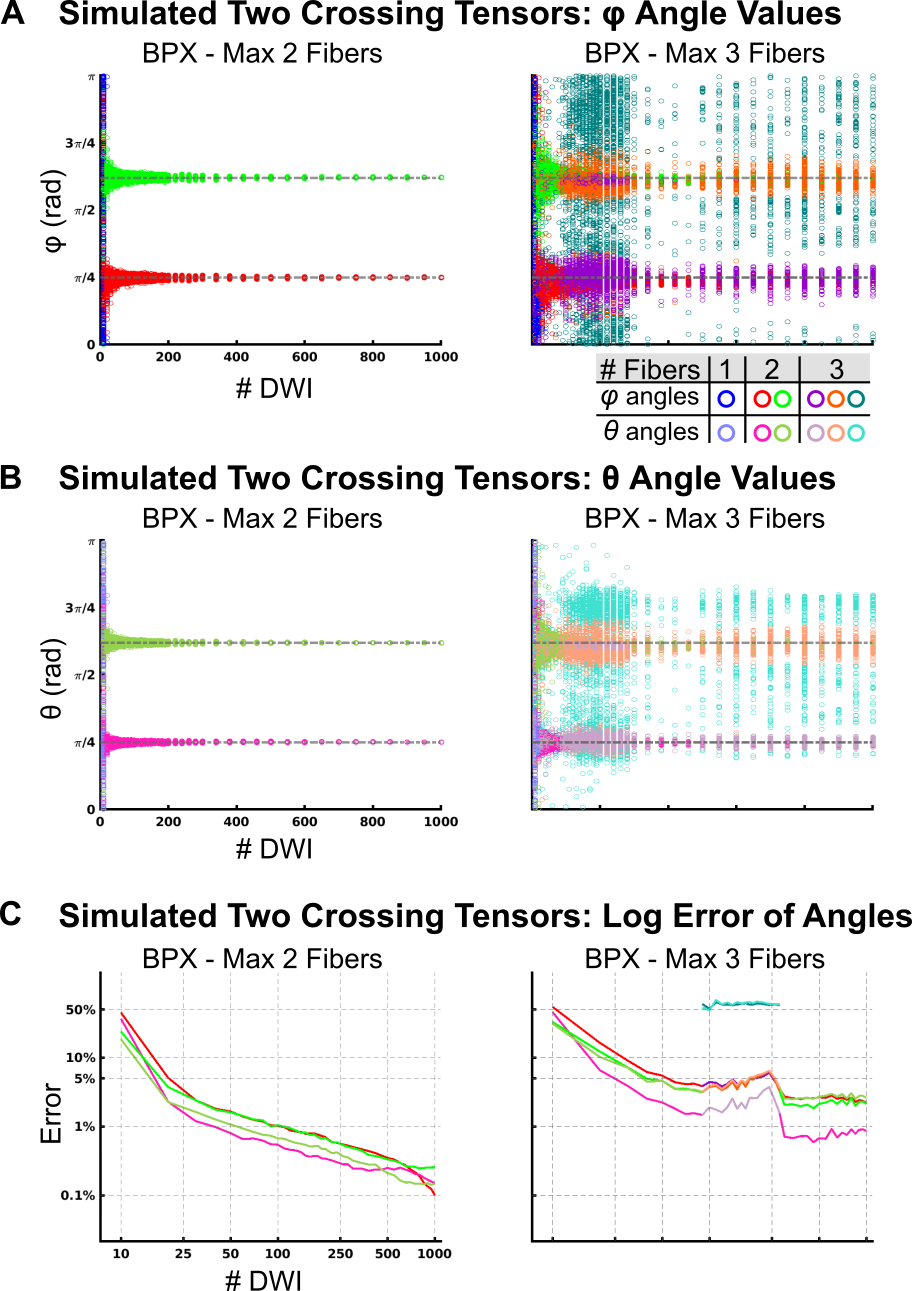
**

**Supplementary Figure 10: Accuracy of BPX in two tensor simulated data with max 3 fibers**

The tensors were oriented such that they were perpendicular to each other. The first tensor had larger weighting equal to 60% of the signal. Rician noise was added for an SNR = 50. (A) φ angle estimations by BedpostX (BPX), with max 2 or three fibers. Open circles represent the results obtained by repeated permutation sampling. Same color legend for all data panels. Permutations that resulted in a single fiber direction are plotted in blue (φ). Permutations that resulted in two fibers are plotted in red (φ) and green (φ). Permutations that resulted in three fibers are plotted in purple (φ), orange (φ), teal (φ). (B) θ angle estimations. Permutations are plotted in sky blue (θ) for one fiber, pink (θ) and olive (θ) for two fibers, and lilac (θ), salmon (θ), cyan (θ) for three fibers. (C) Error estimation for BPX with max two or three fibers. Mean error at each subsampling size was calculated, then plotted on a log scale. The same colors as in (A/B) are used and indicate the most frequent number of fibers estimated at each subsampling size.


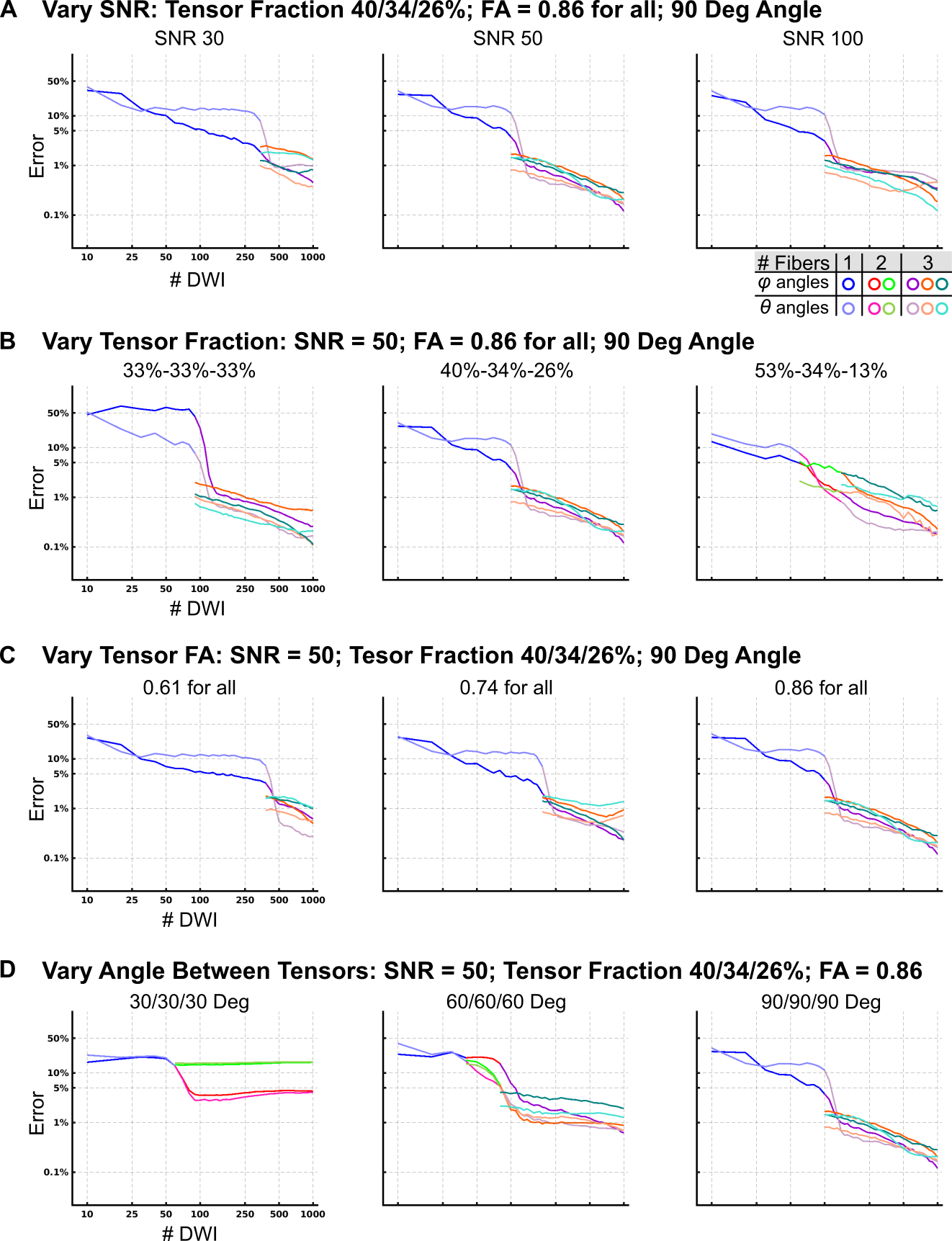


**Supplementary Figure 11: Accuracy of BaMM in variety of three tensor simulated data**

(A) Error estimation by BaMM for three crossing tensors at varying SNR. Mean error at each subsampling size was calculated, then plotted as the log of error. Plots are colored by the most frequent number of fibers estimated: subsamples with a single fiber direction are plotted in blue/sky blue, two fibers plotted red/pink and green/olive, 3 fibers are plotted in purple/lilac, orange/salmon, teal/cyan. (B) Vary tensor Fraction. (C) Vary tensor FA. (D) Vary angle between tensors.

**
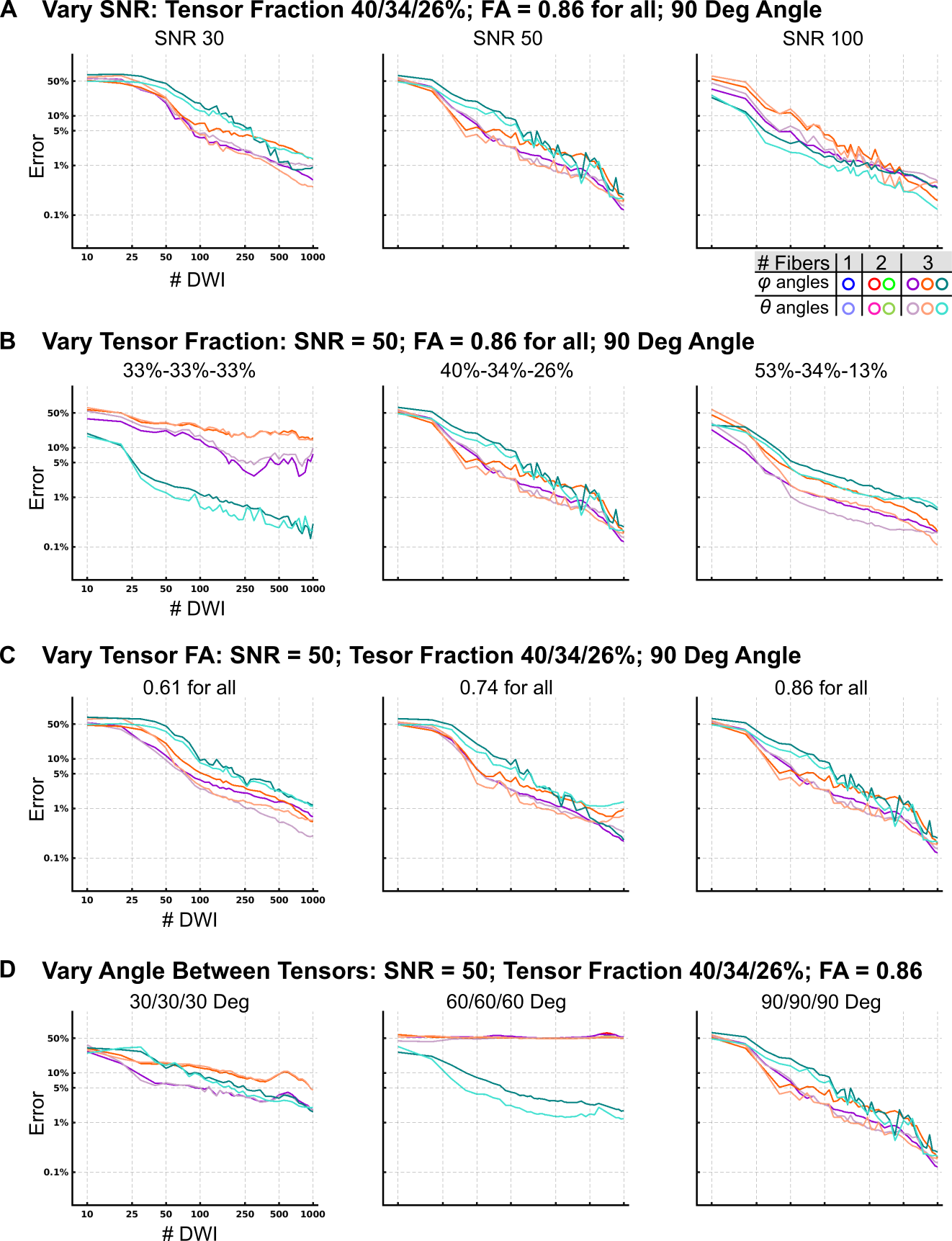
**

**Supplementary Figure 12: Accuracy of BPX in variety of three tensor simulated data**

(A) Error estimation by BPX for three crossing tensors at varying SNR. Mean error at each subsampling size was calculated, then plotted as the log of error. Plots are colored by the most frequent number of fibers estimated: subsamples with a single fiber direction are plotted in blue/sky blue, two fibers plotted red/pink and green/olive, 3 fibers are plotted in purple/lilac, orange/salmon, teal/cyan. (B) Vary tensor Fraction. (C) Vary tensor FA. (D) Vary angle between tensors.

**
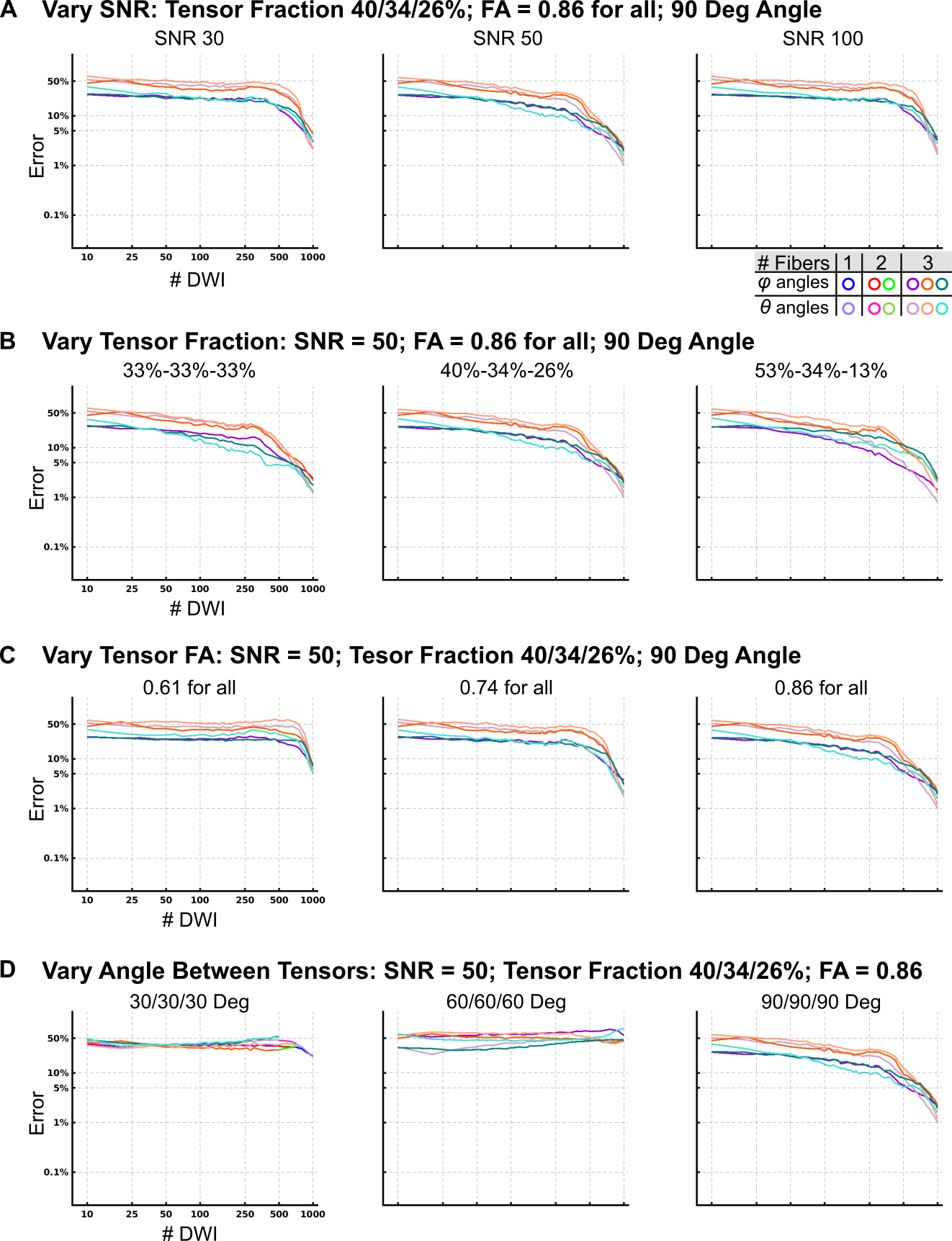
**

**Supplementary Figure 13: Accuracy of CSA-QBI in variety of three tensor simulated data**

(A) Error estimation by CSA-QBI for three crossing tensors at varying SNR. Mean error at each subsampling size was calculated, then plotted as the log of error. Plots are colored by the most frequent number of fibers estimated: subsamples with a single fiber direction are plotted in blue/sky blue, two fibers plotted red/pink and green/olive, 3 fibers are plotted in purple/lilac, orange/salmon, teal/cyan. (B) Vary tensor Fraction. (C) Vary tensor FA. (D) Vary angle between tensors.

**
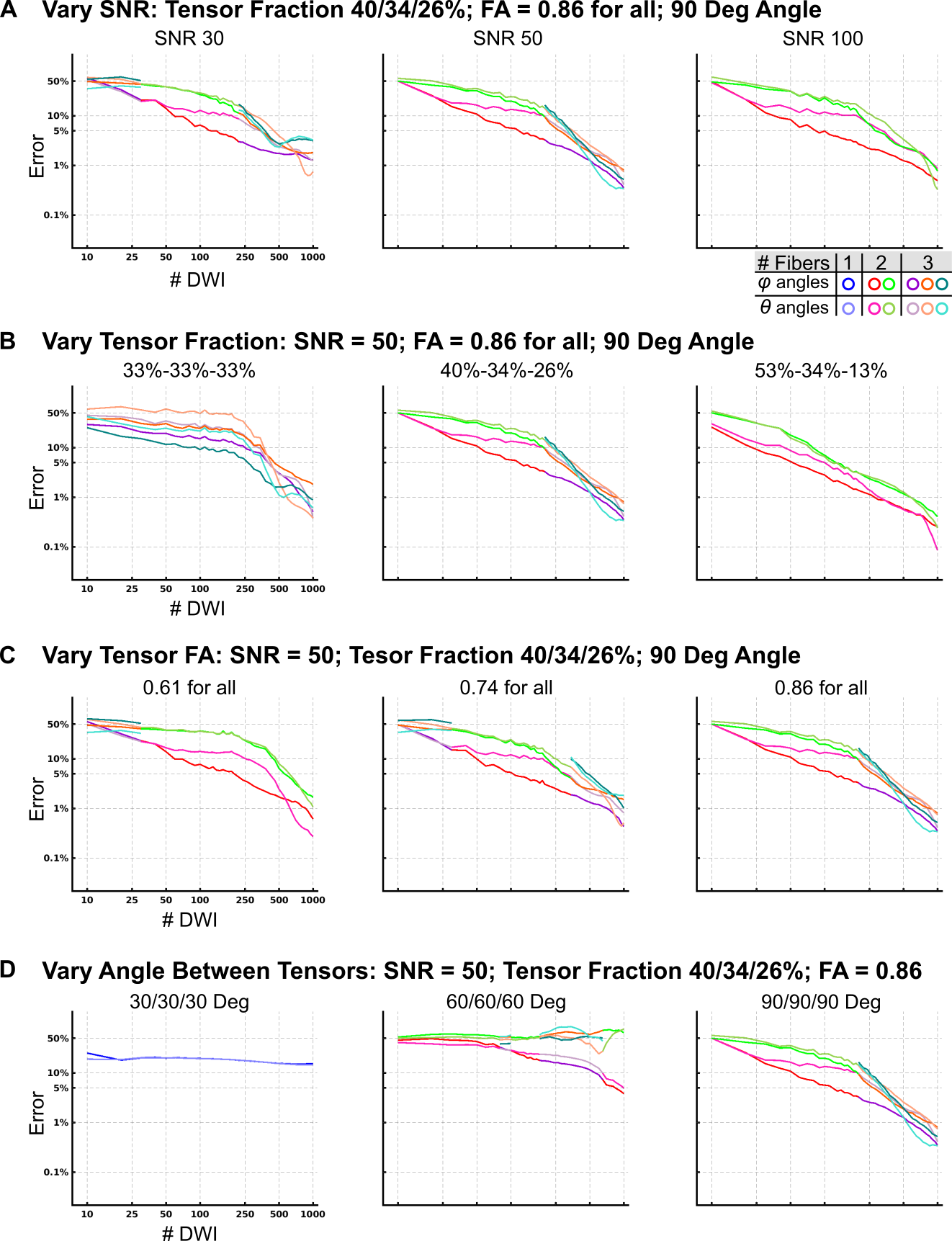
**

**Supplementary Figure 14: Accuracy of CSD in variety of three tensor simulated data**

(A) Error estimation by CSD for three crossing tensors at varying SNR. Mean error at each subsampling size was calculated, then plotted as the log of error. Plots are colored by the most frequent number of fibers estimated: subsamples with a single fiber direction are plotted in blue/sky blue, two fibers plotted red/pink and green/olive, 3 fibers are plotted in purple/lilac, orange/salmon, teal/cyan. (B) Vary tensor Fraction. (C) Vary tensor FA. (D) Vary angle between tensors.

**
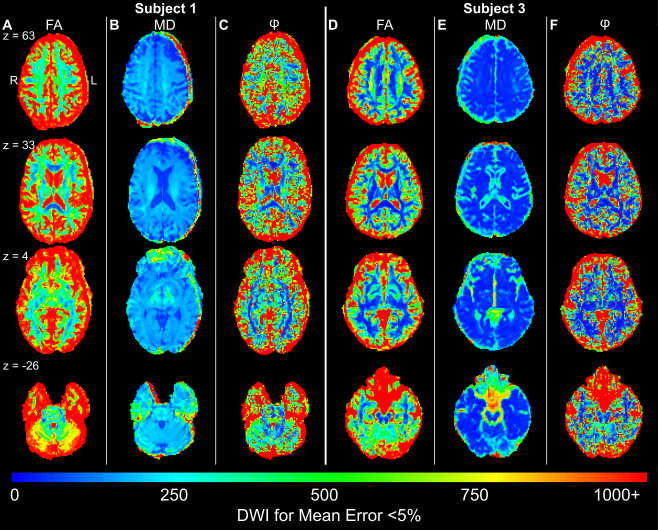
**

**Supplementary Figure 15: LLS whole-brain reliability map for Mean Error < 5%, Subject 1 & and Subject 3**

(A) The color scale shows the number of DWI measurements needed to achieve a voxel-wise error less than 5% in FA in Subject 1. Error is calculated relative to the mean FA found using the entire sample. Results for (B) MD, and (C) angle φ in Subject 1 are shown. For subject 3, (D) FA, (E) MD, (F) angle φ.


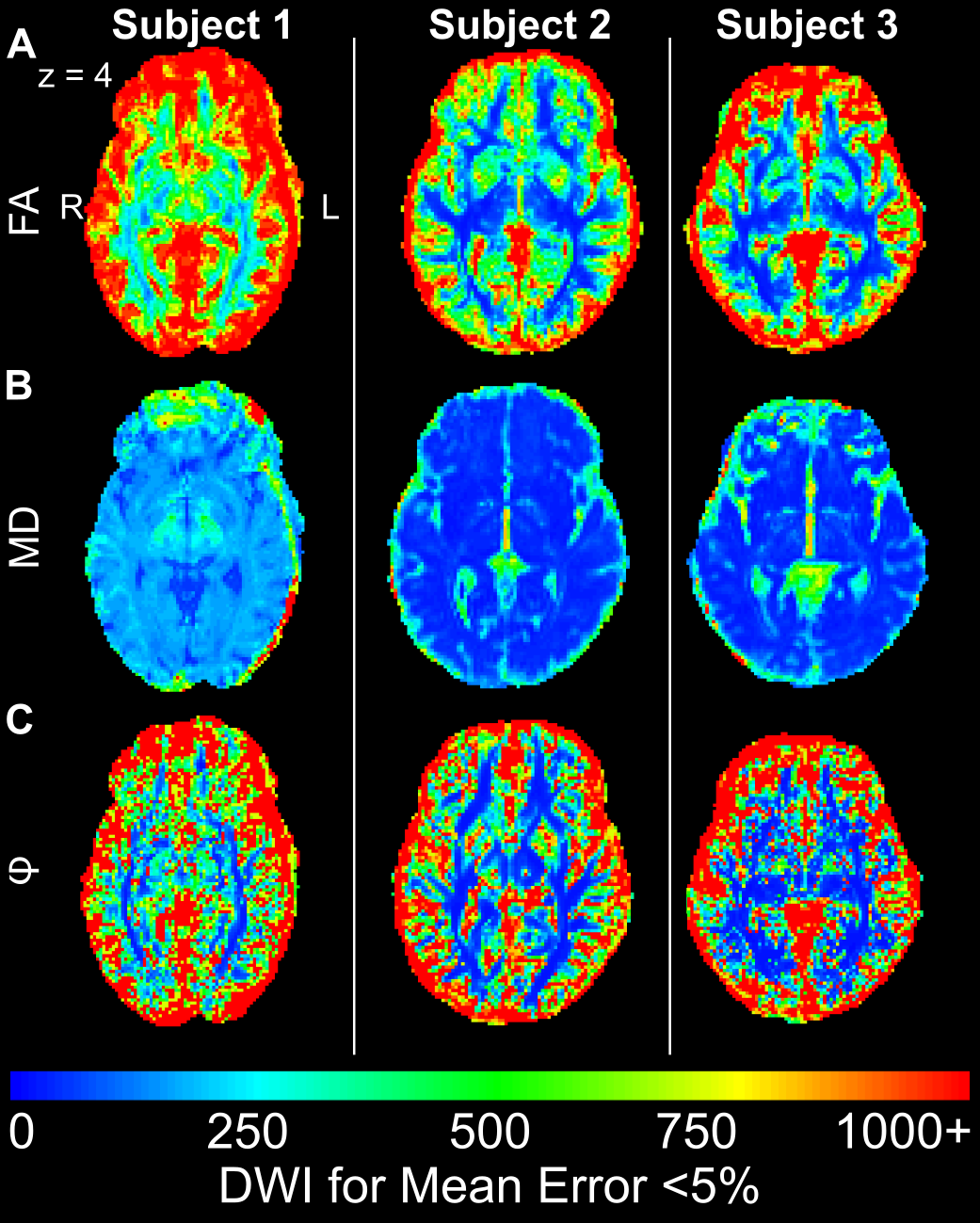


**Supplementary Figure 16: Single-Slice Subject Comparison of LLS Full Brain Reliability**

As in Figure 6 and S15, slice z=4 across subjects for easy inter-individual comparison. (A) The color scale shows the number of DWI measurements needed to achieve a voxel-wise error less than 5% in FA in all subjects. Error is calculated relative to the mean FA found using the entire sample. Results for (B) MD, and (C) angle φ are shown.


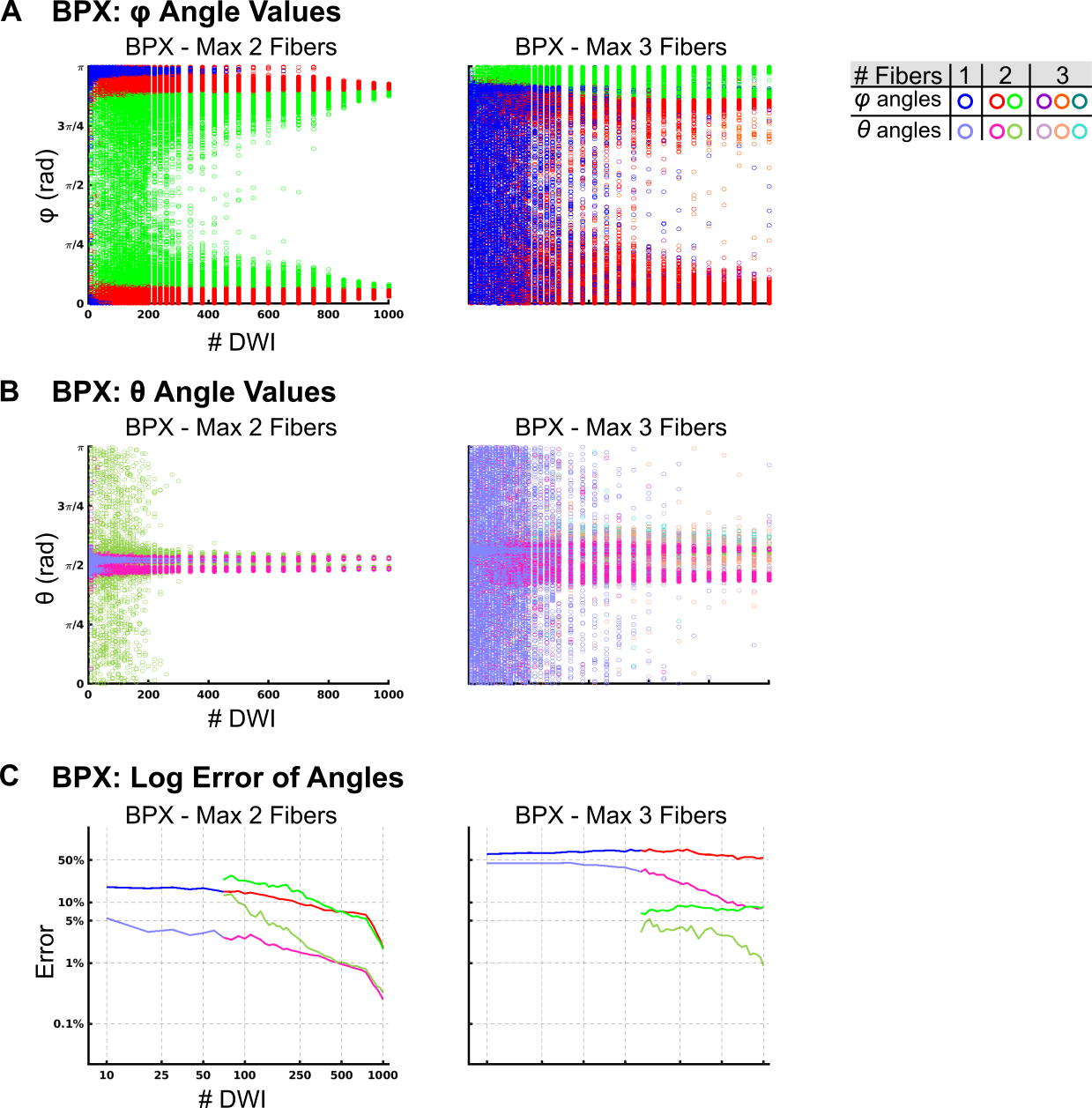


**Supplementary Figure 17: Reliability of BPX Subject 2 Corpus Callosum with max 3 fibers**

The same ROI as in figure 7 in subject 2 is analyzed. (A) φ Angle estimations by BedpostX (BPX) with max two or three fibers. (B) θ Angle estimations by BPX with max two or three fibers. (C) Log Error estimation by BPX with two or three fibers. Error is calculated relative to the mean φ or θ found using the entire sample.

**
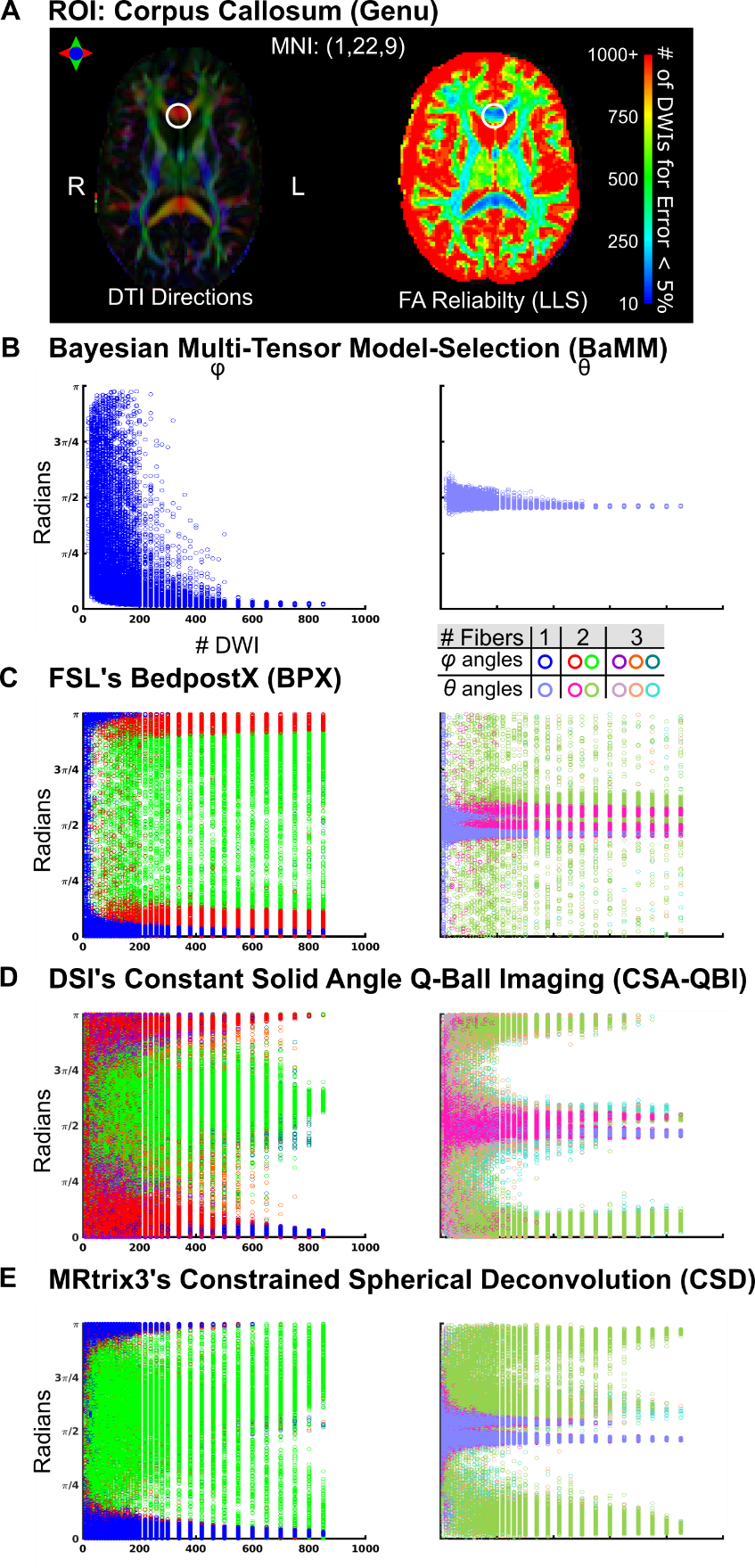
**

**Supplementary Figure 18: Reliability of Diffusion Measures in the Genu of the Corpus Callosum, Subject 1**

(A) The locus of the analyzed voxel (MNI: 1, 22, 9) is marked with a circle. LLS FA reliability map as in Figure 6A. (B) Angle estimations by Bayesian Multi-tensor Model-selection (BaMM). (C) BedpostX (BPX). (D) Constant Solid Angle Q-Ball Imaging (CSA-QBI). (E) Constrained Spherical Deconvolution (CSD)


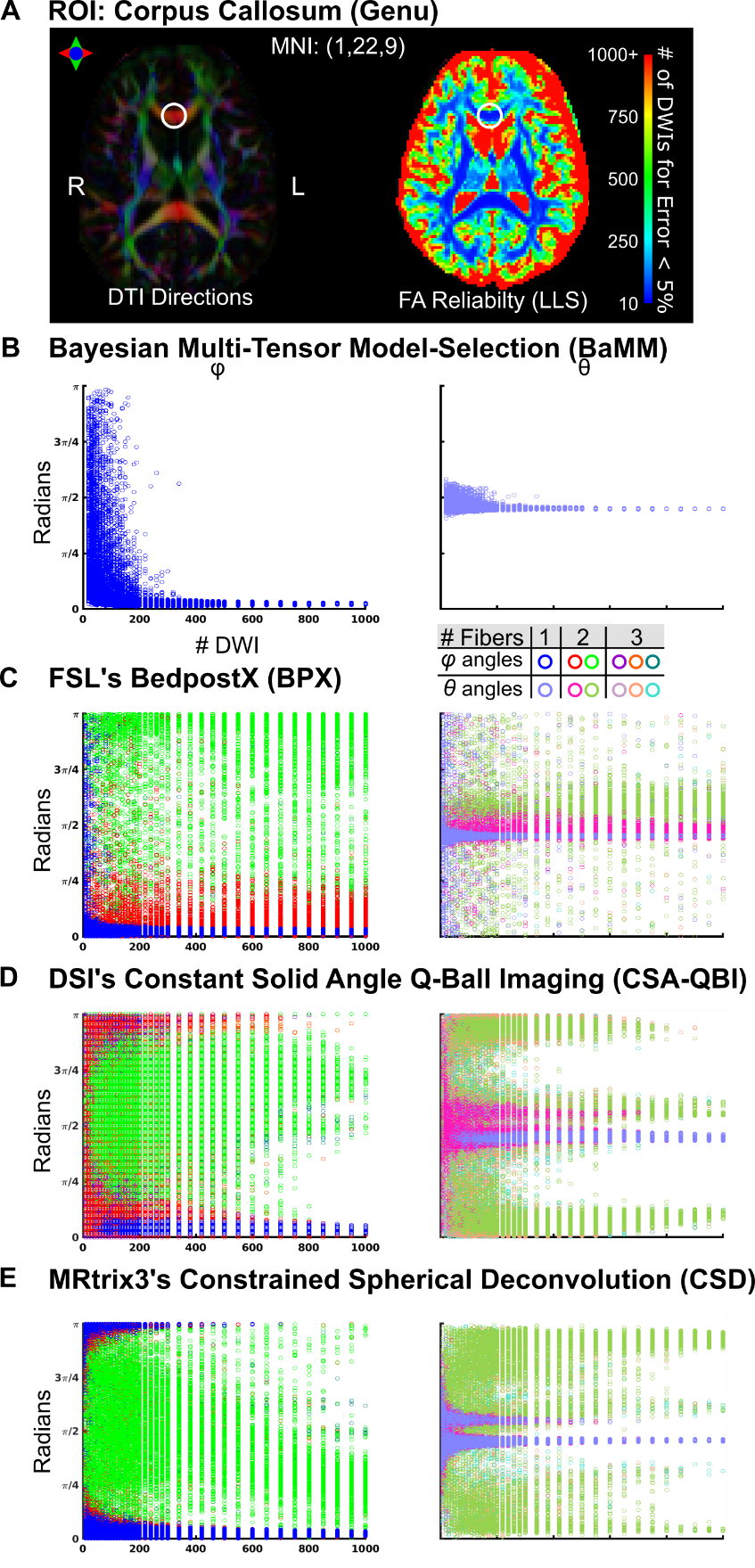


**Supplementary Figure 19: Reliability of Diffusion Measures in the Genu of the Corpus Callosum, Subject 3**

(A) The locus of the analyzed voxel (MNI: 1, 22, 9) is marked with a circle. LLS FA reliability map as in Figure 6A. (B) Angle estimations by Bayesian Multi-tensor Model-selection (BaMM). (C) BedpostX (BPX). (D) Constant Solid Angle Q-Ball Imaging (CSA-QBI). (E) Constrained Spherical Deconvolution (CSD)


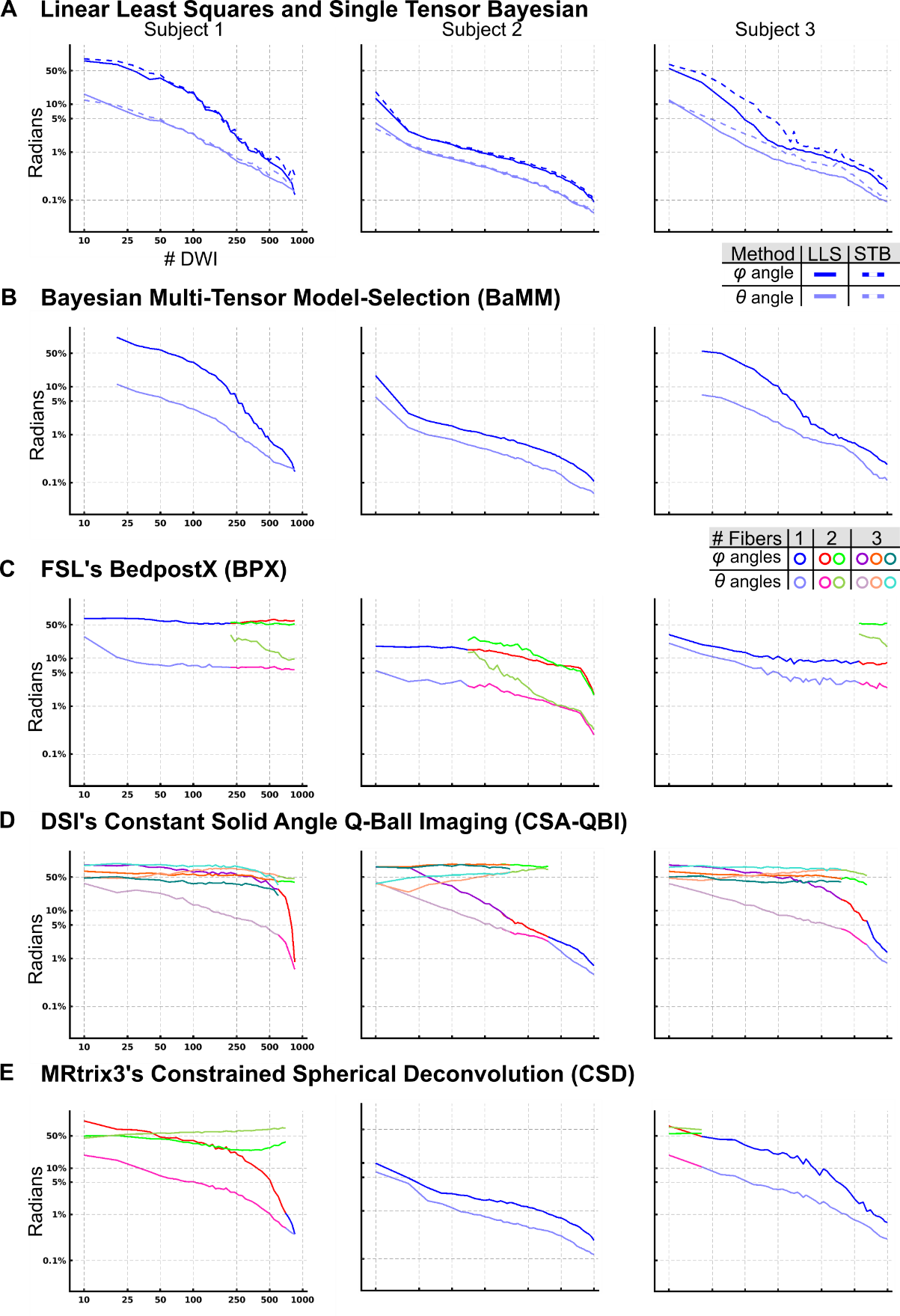


**Supplementary Figure 20: Error Estimations in the Genu of the Corpus Callosum**

(A) Log Error of angle estimation by LLS and STB. Error is calculated relative to the mean φ or θ found by each method using the entire sample. (B) Bayesian Multi-tensor Model-selection (BaMM). (C) BedpostX (BPX). (D) Constant Solid Angle Q-Ball Imaging (CSA-QBI). (E) Constrained Spherical Deconvolution (CSD)


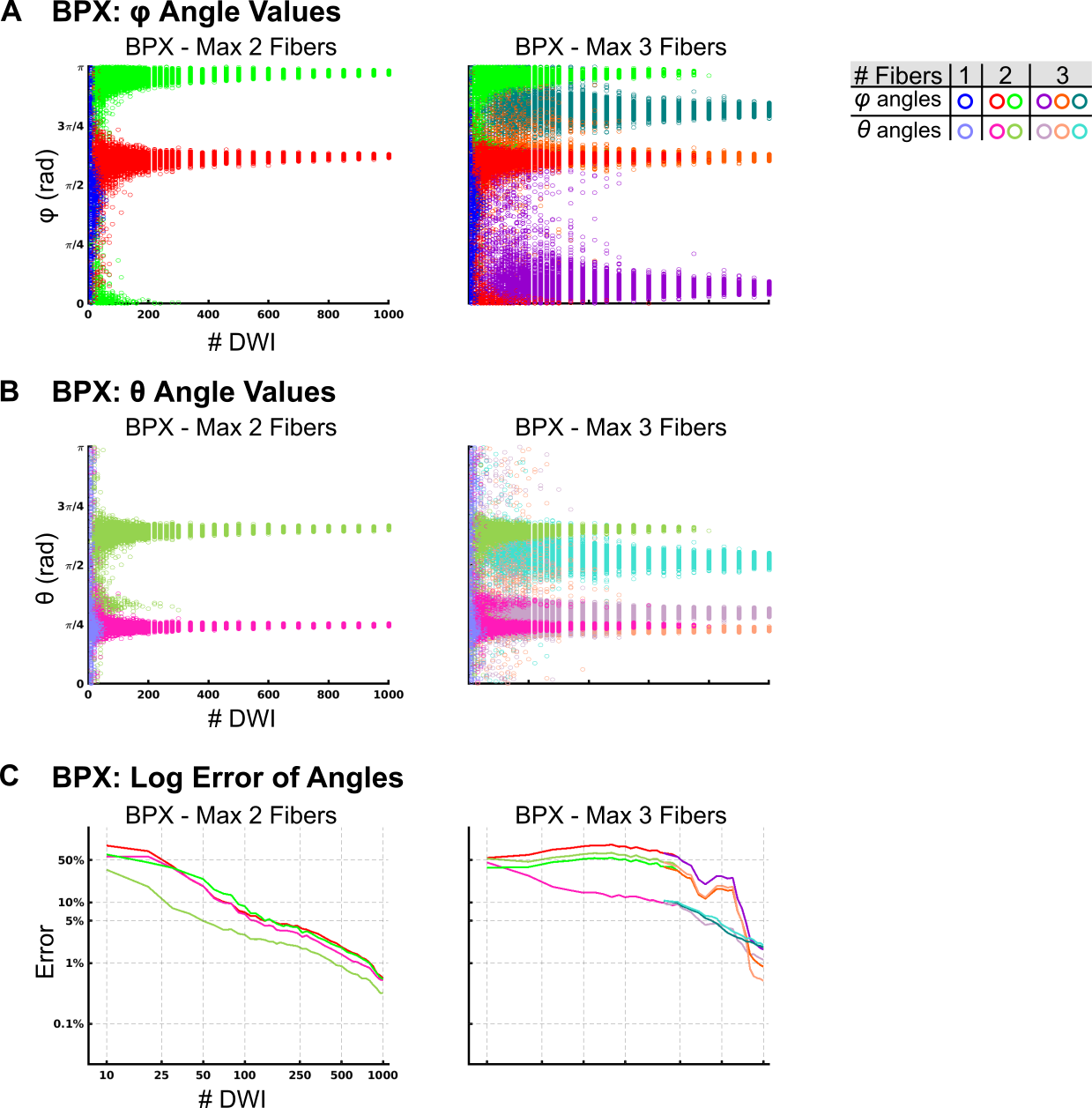


**Supplementary Figure 21: Reliability of BPX Subject 2 Left Frontal White Matter with max 3 fibers**

The same ROI as in figure 8 in subject 2 is analyzed. (A) φ Angle estimations by BedpostX (BPX) with max two or three fibers. (B) θ Angle estimations by BPX with max two or three fibers. (C) Log Error estimation by BPX with two or three fibers. Error is calculated relative to the mean φ or θ found using the entire sample.

**
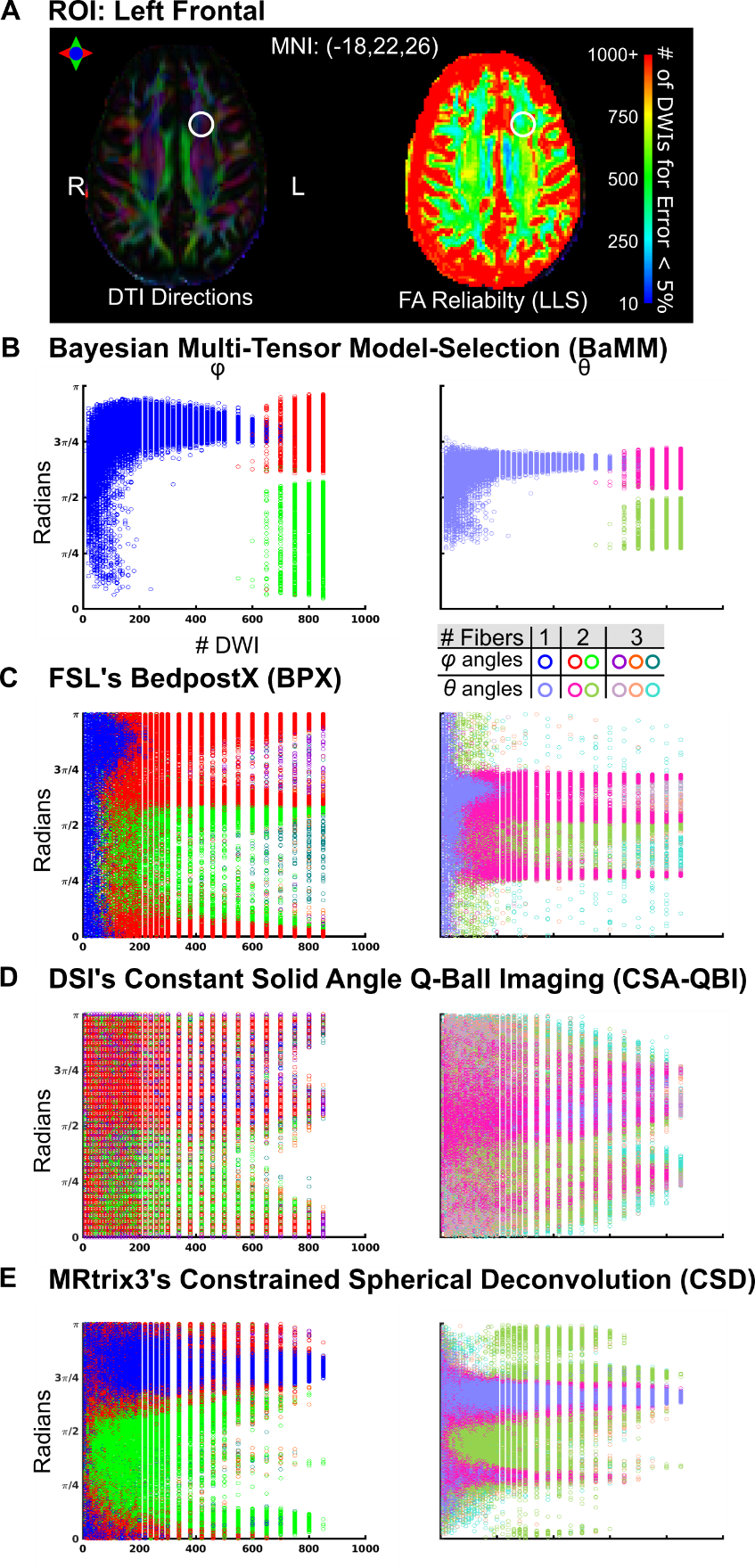
**

**Supplementary Figure 22: Reliability of Diffusion Measures in the Left Frontal White Matter, Subject 1**

(A) The locus of the analyzed voxel (MNI: 18, 22,26) is marked with a circle. LLS FA reliability map as in Figure 6A. (B) Angle estimations by Bayesian Multi-tensor Model-selection (BaMM). (C) BedpostX (BPX). (D) Constant Solid Angle Q-Ball Imaging (CSA-QBI). (E) Constrained Spherical Deconvolution (CSD)


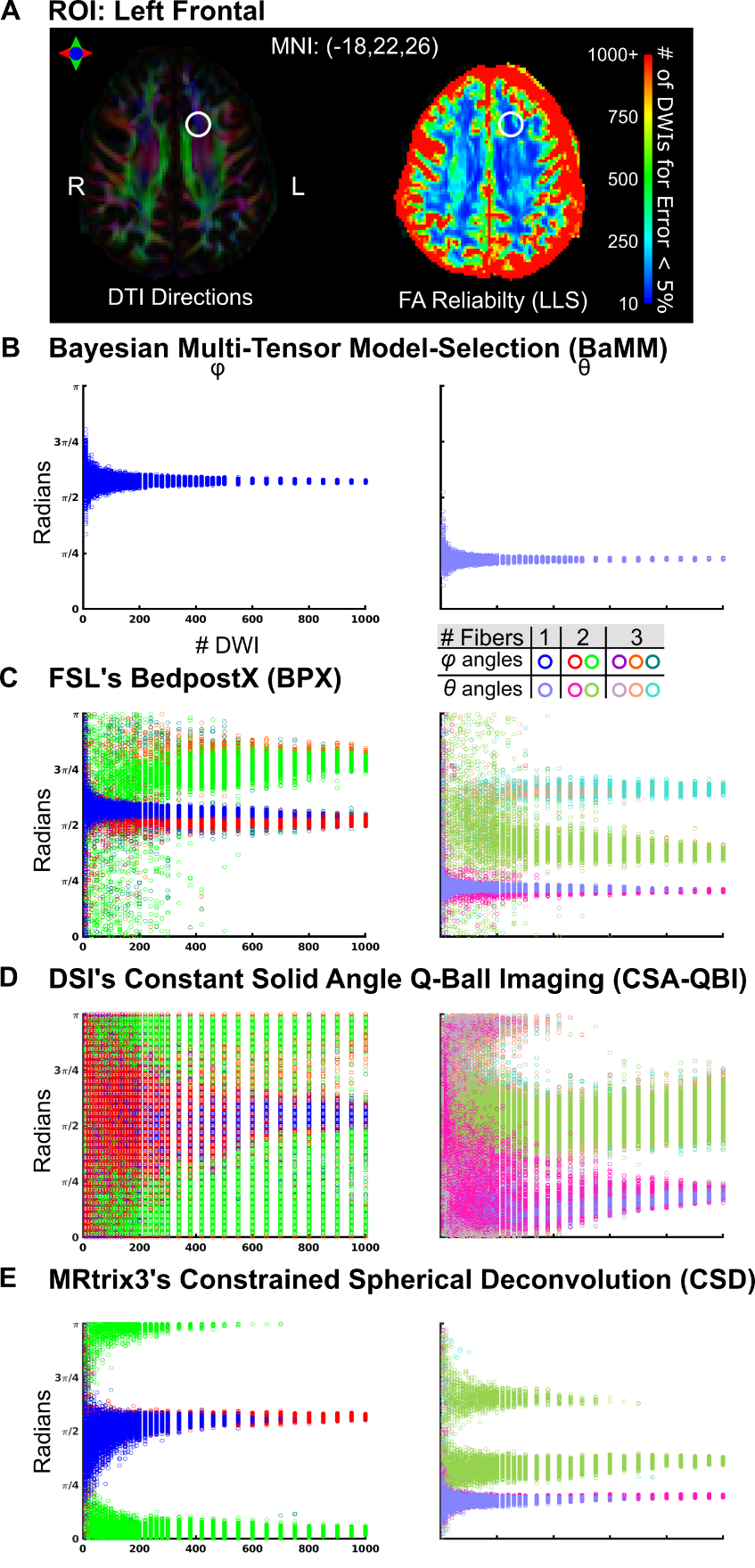


**Supplementary Figure 23: Reliability of Diffusion Measures in the Left Frontal White Matter, Subject 3**

(A) The locus of the analyzed voxel (MNI: 18, 22,26) is marked with a circle. LLS FA reliability map as in Figure 6A. (B) Angle estimations by Bayesian Multi-tensor Model-selection (BaMM). (C) BedpostX (BPX). (D) Constant Solid Angle Q-Ball Imaging CSA- (QBI). (E) Constrained Spherical Deconvolution (CSD)


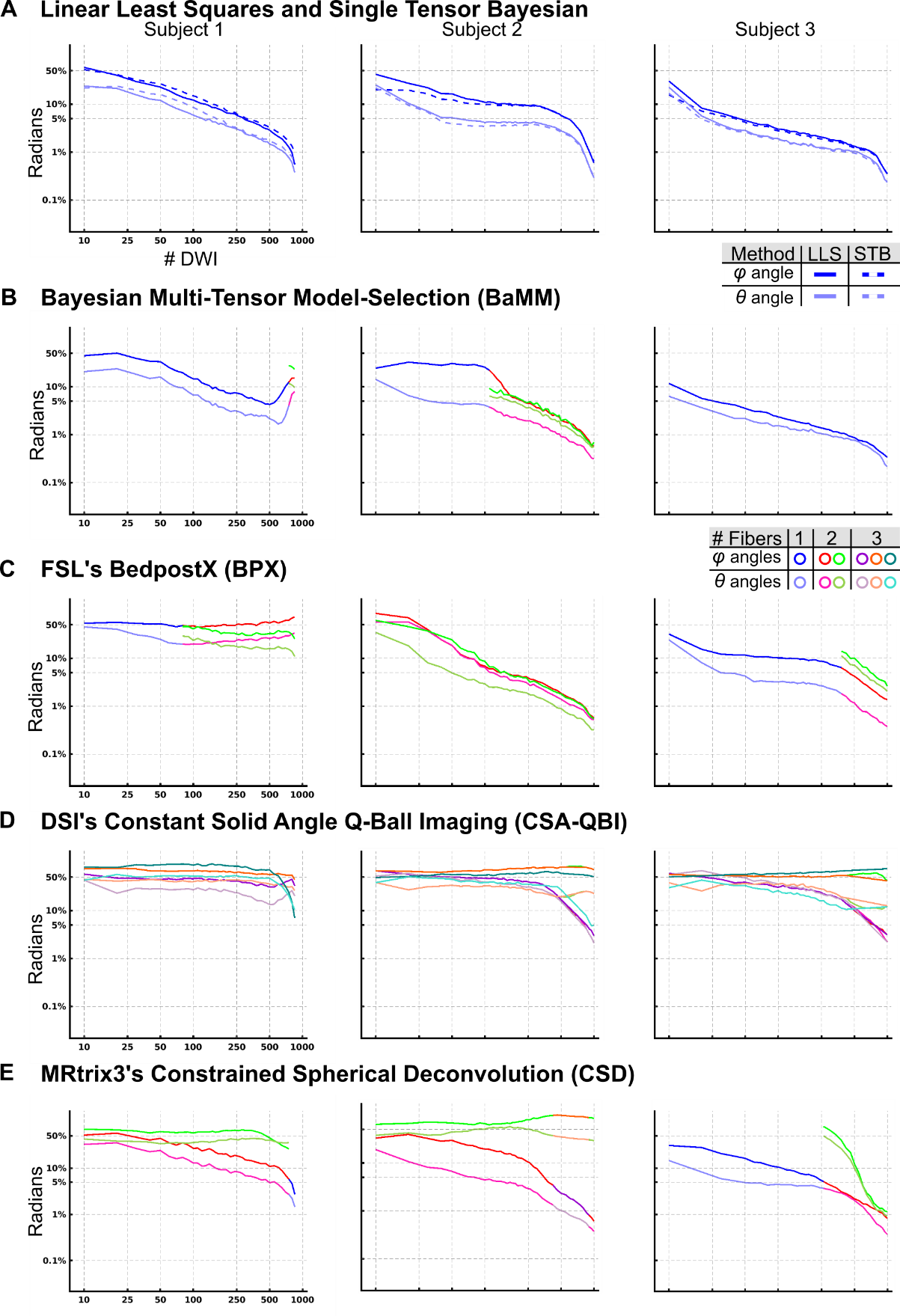


**Supplementary Figure 24: Error Estimations in the Left Frontal White Matter**

(A) Log Error of angle estimation by LLS and STB. Error is calculated relative to the mean φ or θ found by each method using the entire sample. (B) Bayesian Multi-tensor Model-selection (BaMM). (C) BedpostX (BPX). (D) Constant Solid Angle Q-Ball Imaging (CSA-QBI). (E) Constrained Spherical Deconvolution (CSD)

**
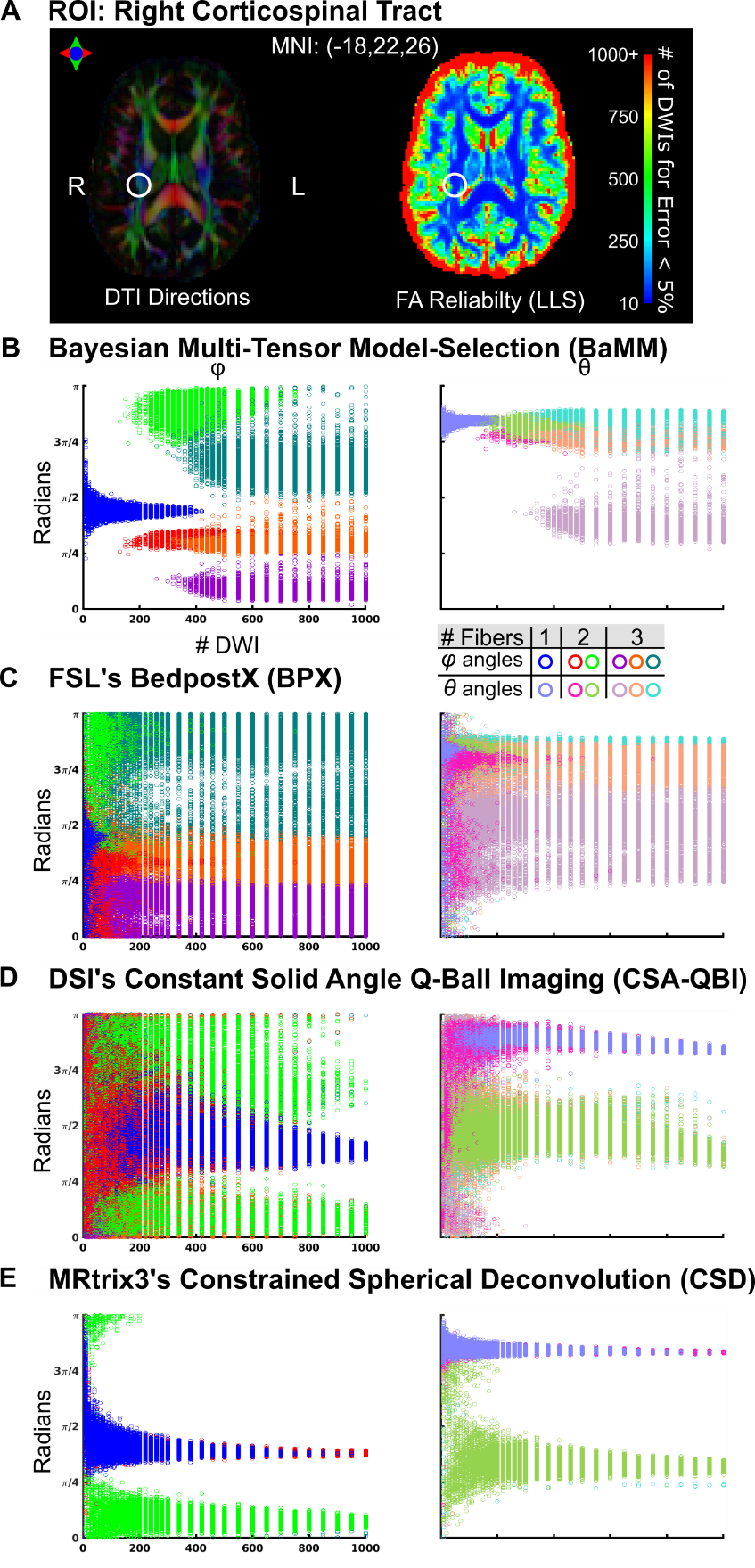
**

**Supplementary Figure 25: Reliability of Diffusion Measures in Right Corticospinal Tract, Subject 2**

(A) The locus of the analyzed voxel (MNI: -22, -19, 11) is marked with a circle. LLS FA reliability map as in Figure 6A. (B) Angle estimations by Bayesian Multi-tensor Model-selection (BaMM). (C) BedpostX (BPX). (D) Constant Solid Angle Q-Ball Imaging (CSA-QBI). (E) Constrained Spherical Deconvolution (CSD)


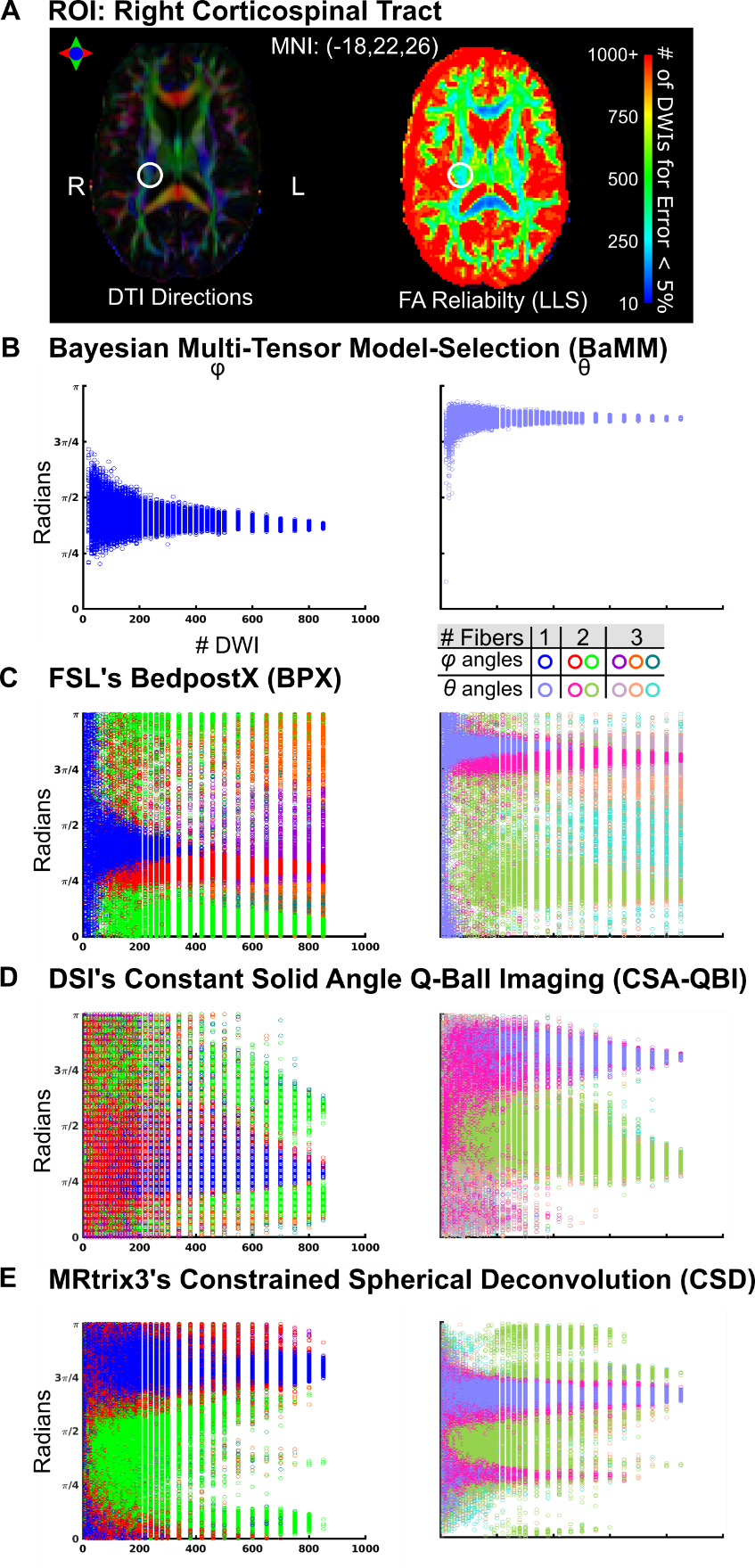


**Supplementary Figure 26: Reliability of Diffusion Measures in Right Corticospinal Tract, Subject 1**

(A) The locus of the analyzed voxel (MNI: -22, -19, 11) is marked with a circle. LLS FA reliability map as in Figure 6A. (B) Angle estimations by Bayesian Multi-tensor Model-selection (BaMM). (C) BedpostX (BPX). (D) Constant Solid Angle Q-Ball Imaging (CSA-QBI). (E) Constrained Spherical Deconvolution (CSD)

**
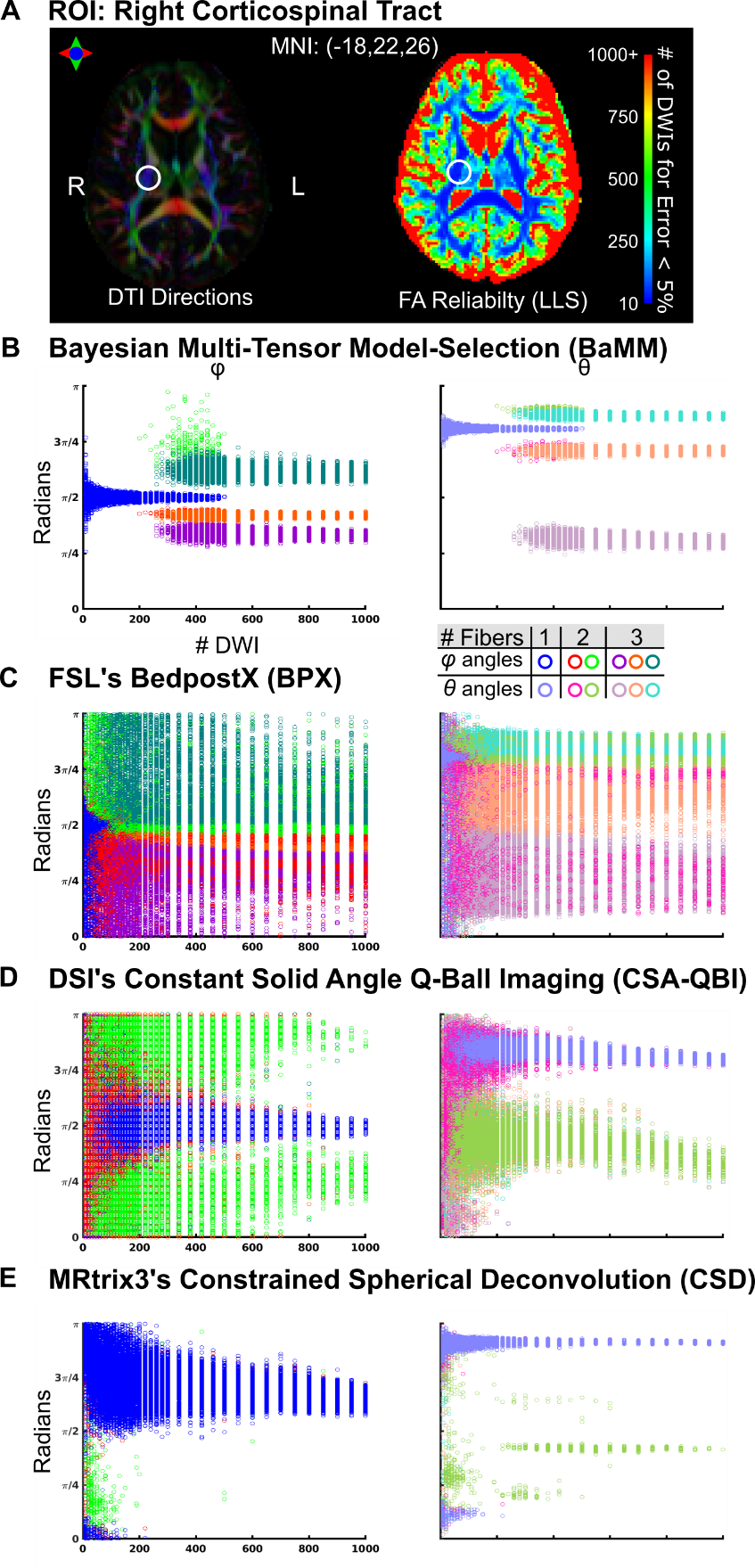
**

**Supplementary Figure 27: Reliability of Diffusion Measures in Right Corticospinal Tract, Subject 3**

(A) The locus of the analyzed voxel (MNI: -22, -19, 11) is marked with a circle. LLS FA reliability map as in Figure 6A. (B) Angle estimations by Bayesian Multi-tensor Model-selection (BaMM). (C) BedpostX (BPX). (D) Constant Solid Angle Q-Ball Imaging (CSA-QBI). (E) Constrained Spherical Deconvolution (CSD)

**
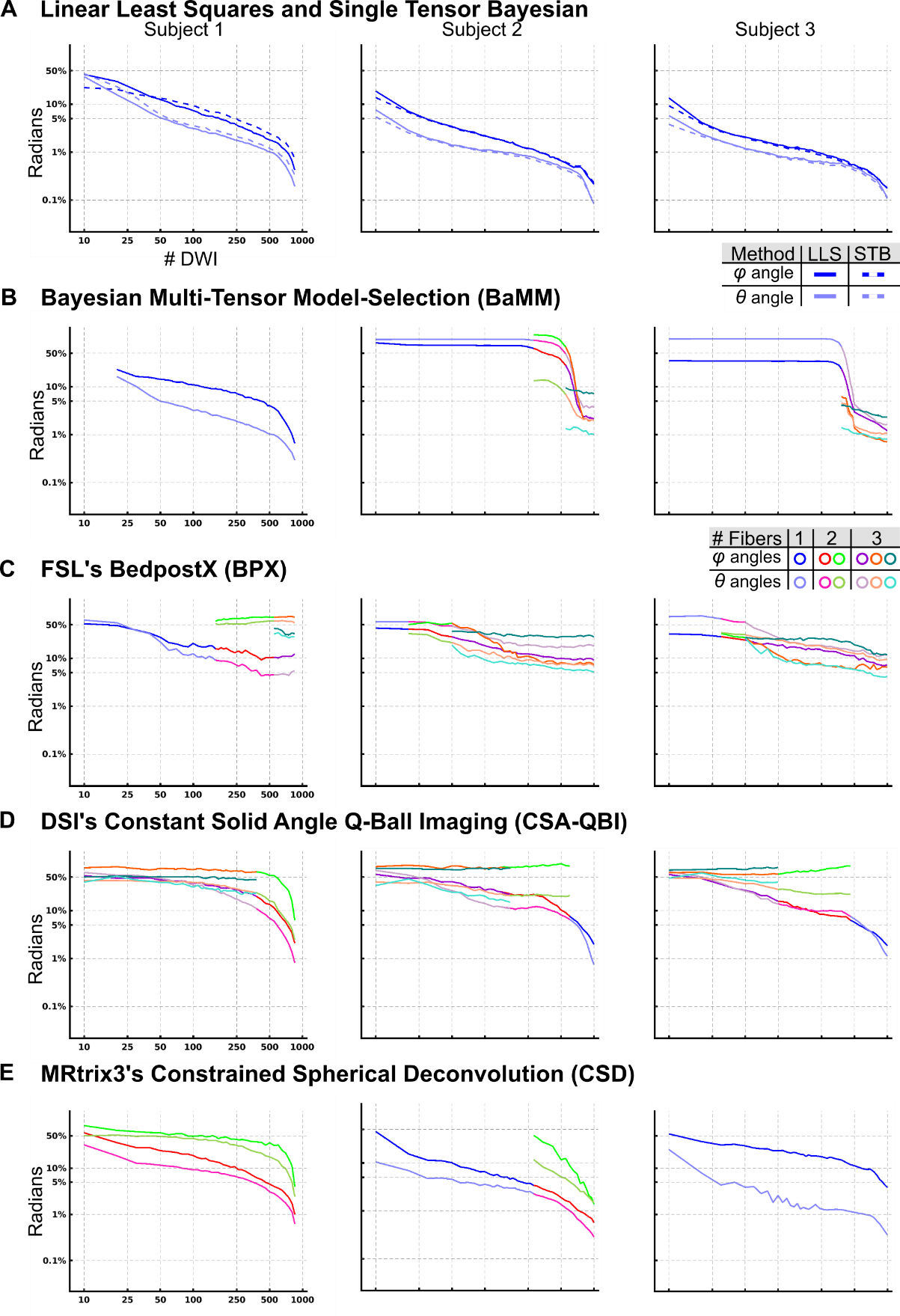
**

**Supplementary Figure 28: Error Estimations in Right Corticospinal Tract**

(A) Log Error of angle estimation by LLS and STB. Error is calculated relative to the mean φ or θ found by each method using the entire sample. (B) Bayesian Multi-tensor Model-selection (BaMM). (C) BedpostX (BPX). (D) Constant Solid Angle Q-Ball Imaging (CSA-QBI). (E) Constrained Spherical Deconvolution (CSD)

**
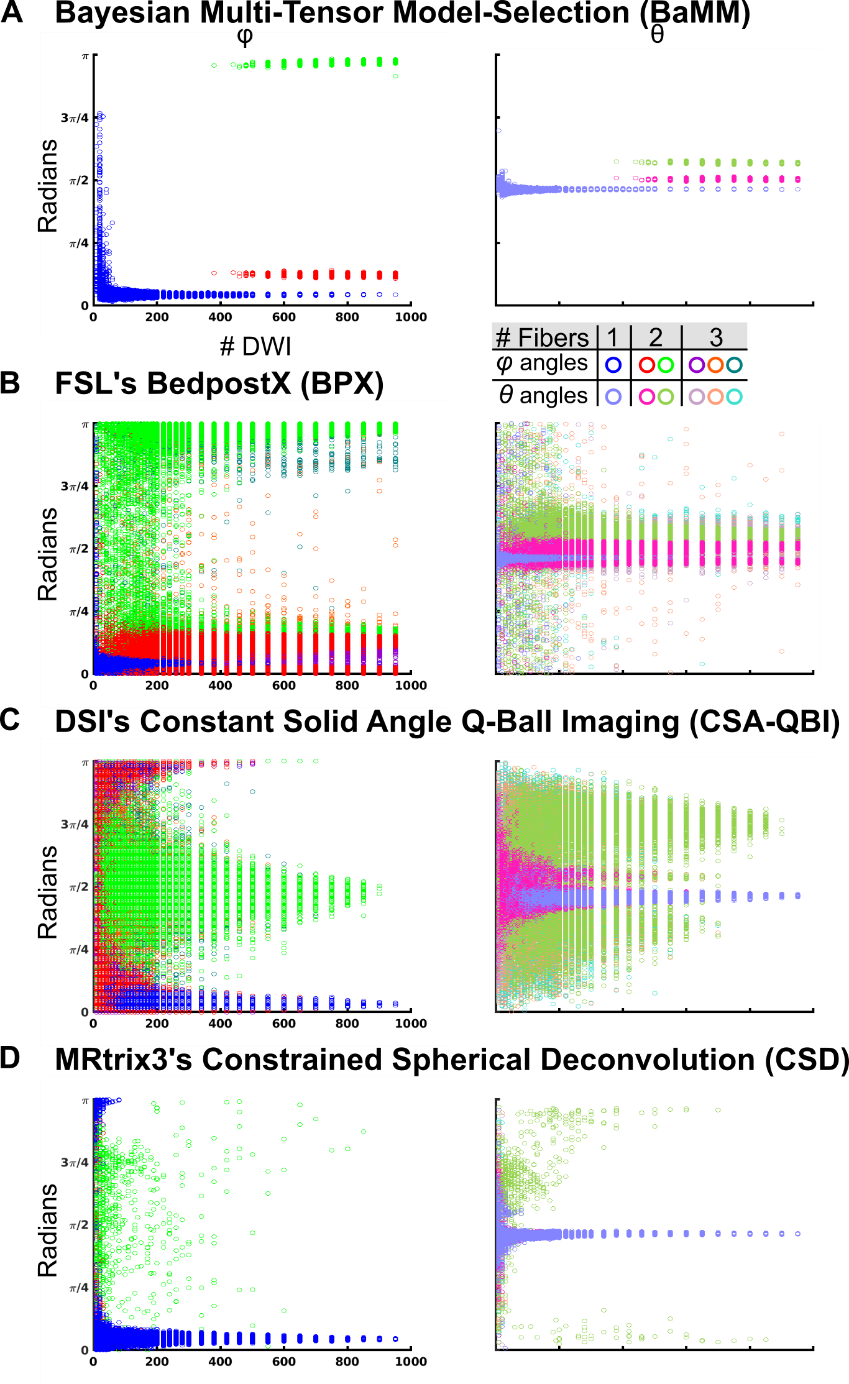
**

**Supplementary Figure 29: Reliability of Diffusion Measures in the Genu of the Corpus Callosum with SS-HAR, Subject 1**

Subject 1 was rescanned with a high-angular resolution sequence. Repeated sampling output in figure S18. (A) Angle estimations by Bayesian Multi-tensor Model-selection (BaMM). (B) BedpostX (BPX). (C) Constant Solid Angle Q-Ball Imaging (CSA-QBI). (D) Constrained Spherical Deconvolution (CSD)
